# Long-term expansion of basal cells and the novel differentiation methods identify mechanisms for switching Claudin expression in normal epithelia

**DOI:** 10.1038/s41598-025-95463-3

**Published:** 2025-04-09

**Authors:** Akihito Inoko, Norihito Soga, Minako Suzuki, Tohru Kiyono, Junichi Ikenouchi, Takahiro Kojima, Yoshikatsu Sato, Daisuke Saito, Tatsuo Miyamoto, Naoki Goshima, Hideaki Ito, Kenji Kasai

**Affiliations:** 1 Department of Pathology, Aichi Medical University School of Medicine, Nagakute, Japan; 2 Department of Urology, Aichi Cancer Center Hospital, Nagoya, Japan; 3 Project for Prevention of HPV-Related Cancer, Exploratory Oncology Research and Clinical Trial Center, National Cancer Center, Chiba, Japan; 4 Department of Biology, Faculty of Sciences, Kyushu University, Fukuoka, Japan; 5 Institute of Transformative Bio-Molecules (WPI-ITbM), Nagoya University, Nagoya, Japan; 6 Department of Molecular and Cellular Physiology, Yamaguchi University Graduate School of Medicine, Ube, Japan; 7 Molecular Profiling Research Center for Drug Discovery, National Institute of Advanced Industrial Science and Technology (AIST), Tokyo, Japan

**Keywords:** Epithelium, Epithelial-mesenchymal transition (EMT), Claudin, Primary cell culture, Cell differentiation, Basal cell, Tight junctions, Cancer, Cell biology

## Abstract

Epithelia are tightly connected cellular sheets, that shield our body from the external environment. They are continuously maintained by a pooled population of undifferentiated cells through differentiation. However, the maintenance mechanisms remain incompletely understood due to the difficulty of experimentally observing the differentiation process. To address this issue, we developed a culture method for long-term expansion of primary mammary basal cells with a set of compounds, that includes undifferentiated cells. An effective differentiation method regarding Claudin expression was also developed by simply removing compounds. To verify this differentiation-switching technique, we obtained microarray data comparing each differentiation state. Subsequent cellular analysis confirmed key transcription factors in each state: (1) EGR1 in undifferentiated basal cells is important for suppressing Claudin expression through maintaining the epithelial-mesenchymal transition (EMT) transcription factor TWIST1, (2) ELF3 in differentiated cells is important for actin organization and subsequent Claudin localization at the cell-cell border, that corresponds to the amount of GRHL3, an actin organizer. Their relevance was also observed in tissues and organoids. In summary, we present an effective tool for verifying molecular mechanisms that determine Claudin status in normal basal cell differentiation, that would be beneficial in epithelial cell biology, cancer biology, physiology, and regeneration research.

**Supplementary Information:**

The online version contains supplementary material available at 10.1038/s41598-025-95463-3.

## Introduction

Epithelia are cellular sheets that tightly adhere to each other and protect organisms from their external environments^1^. Loss of cell-cell adhesion is involved in pathological processes such as inflammation, infection, and cancer progression^2,3^. Many functional studies have identified the key molecules localizing at cell-cell adhesion, such as E-cadherin^4,5^, desmosomal cadherin^6^, and claudins^7,8^. In particular, claudins at tight junctions act as the selective paracellular barrier, maintaining the distinct internal environment of each organ^9,10^. The maintenance of barrier function requires epithelial tissues of constant renewal by stem cell differentiation. Indeed, in vivo studies have shown that stem cells facilitate epithelial tissue repair upon injury or transplantation^11–13^.

Epithelia in histology can be classified into single (simple)- and multilayered (stratified or bilayered)- types^12^. Basal cells in multilayered epithelia generally express the truncated isoform of p63 (ΔNp63, hereafter referred to as p63) and are thought to contain tissue-specific stem/progenitor cells (encompassing both the original gland stem cells and several progenitor cells derived from these, hereafter referred to as stem/progenitor cells)^14–16^. The mammary gland is bilayered, with two major cell lineages lining each layer: (1) p63-positive basal cells, some of which are considered mammary stem/progenitor cells and (2) upper luminal epithelial cells covering the ducts^12^. The potency/fate of mammary stem/progenitor cells depends on the surrounding tissue environment. In adult tissue, they exist in each layer and are unipotent. However, mammary basal stem/progenitor cells transplanted into fat pad acquire multipotency and can regenerate a new bilayered gland^17^. The prostate shows similar plasticity^18^. Additionally, cultured mammary basal cells form a bilayer when transferred into a 3D organoid condition^19^. Collectively, basal stem/progenitor cells in bilayered epithelia can differentiate basal myoepithelial and luminal epithelial cells, depending on niche signals.

In this regard, the Blanpain lab experimentally investigated heterotypic cell-cell communication using organoids and mouse models^20^. They demonstrated that luminal cells suppress the multipotency of basal cells by secreting TNF, whereas the loss of luminal cells activates Notch, Wnt, and EGFR signals in basal cells, thereby stimulating their multipotency. However, the intracellular mechanisms governing how basal stem/progenitor cells differentiate into luminal epithelial cells remain incompletely understood.

In human body, mammary gland differentiation is a complex process that dynamically changes over long-term physiological events, such as puberty, pregnancy, and lactation^21^. To address this, a powerful method was developed to identify in vivo differentiation markers by isolating tissue cells using a cell sorter and/or performing comprehensive transcriptome analysis of the tissue^22,23^. However, such in vivo analyses are primarily observational and provide only limited functional verification of differentiation markers.

Simpler epithelial differentiation models are desired for easy observation and intervention. Organoids are an option for observing multipotency^24,25^. However, the heterogeneity in organoids and the Matrigel used for 3D culture potentially interfere with the analysis of individual cells, thus representing drawbacks^26^. Conventional cell lines and genetically modified mice are mainly restricted to observing post-differentiation functions, such as epithelial barrier functions^27,28^. Labeling stem/progenitor cells in animals requires great effort on the genetic engineering side^21^. Overall, most currently available epithelial models do not provide a convenient means for synchronous cell differentiation, manipulation, and observation of the studied changes.

In contrast, primary epithelial cell culture holds potential as a convenient epithelial differentiation-switching model, owing to the presence of stem/progenitor cells and ease of experimental manipulation. For example, primary keratinocytes undergo epithelial differentiation by upregulating the extracellular calcium concentration^29,30^. Primary keratinocytes and airway stem cells also undergo epithelial differentiation in air-liquid interface culture. However, primary cultures remain immature regarding long-term maintenance and differentiation efficiency. Therefore, our knowledge of how external differentiation-leading factors, such as calcium and air, are “read” by stem cells and how stem cells suppress these signals to maintain stemness, is fragmented.

Therefore, it is necessary to improve the method for expanding and differentiating primary epithelial cells, which is a good option for understanding cell fate-determining signals. The first success was the serial cultivation of human epidermal keratinocytes cocultured with irradiated fibroblasts as feeder cells^31^. Recently, long-term culture of primary epithelia, including epidermis, breast, prostate, and certain cancers, has been made possible through the use of feeder cells and F medium supplemented with the Rho-related protein kinase (ROCK) inhibitor Y-27632^32–35^. Furthermore, inhibition of dual SMAD signaling through the addition of DMH1 and A-83-01 to culture media enabled the feeder-free proliferation of p63-positive basal stem cells for an extended period of time^19^. In particular, combining ROCK inhibition via Y-27632 and dual SMAD inhibition had the greatest effect on airway stem cell proliferation.

These culture methods and other in vivo experiments have gradually elucidated the mechanisms that switch between epithelial stem cell proliferation and differentiation. Indeed, SMAD signaling is crucial for the differentiation of p63-positive airway cultured cells^19^ and is also associated with hair follicle regeneration in vivo^36^. The differentiation of cultured epidermal stem cells is reportedly mediated by the actin cytoskeleton^37^. The Wnt pathway may play an important role in stem cell regeneration and quiescence in various tissues^21^. However, these examples are limited. The diversity of mechanisms controlling normal differentiation is incompletely understood among tissues, particularly regarding epithelial-mesenchymal transition (EMT) and the opposite MET, which are also related to cancer^3,13^. One option is optimizing the long-term expansion methods along each tissue type and developing methods for differentiating them with high efficiency.

In this study, we developed a differentiation-switching system with normal primary human mammary epithelial cells (HMECs), that is composed of two culture methods: (1) a chemical compounds-dependent long-term culture method for expanding basal cells that include cells with differentiation potential, and (2) the differentiation method by removing these compounds; that is rapid, simple, and highly efficient. They are convenient for observing switching differentiation regarding Claudin expression. As proof of principle, we performed a combination of comparative microarray analysis and subsequent cellular analyses, thereby functionally identifying EGR1 and ELF3 as the undifferentiated and differentiated markers, respectively. Moreover, our data suggest that their mechanisms are EMT through TWIST1 and actin organization through GRHL3, respectively. Their relevance was also observed in tissues and organoids. Thus, our culturing approach is useful for simplifying the normal epithelial differentiation process and understanding the molecular mechanisms.

## Results

### YDAC-containing F medium can better expand the mammary basal cell population that can express Claudin after drug withdrawal

ROCK and dual SMAD inhibition by Y-27632, DMH1, and A-83-01 is reportedly conducive to extended p63-positive epithelial primary cell culture^19^. In contrast, the impact of Wnt activators, such as CHIR99021, on prolonged culture is still under debate. Indeed, a low-calcium medium does not require Wnt stimulation for the long-term expansion of p63-positive epithelium^38^.

**Fig. 1:**
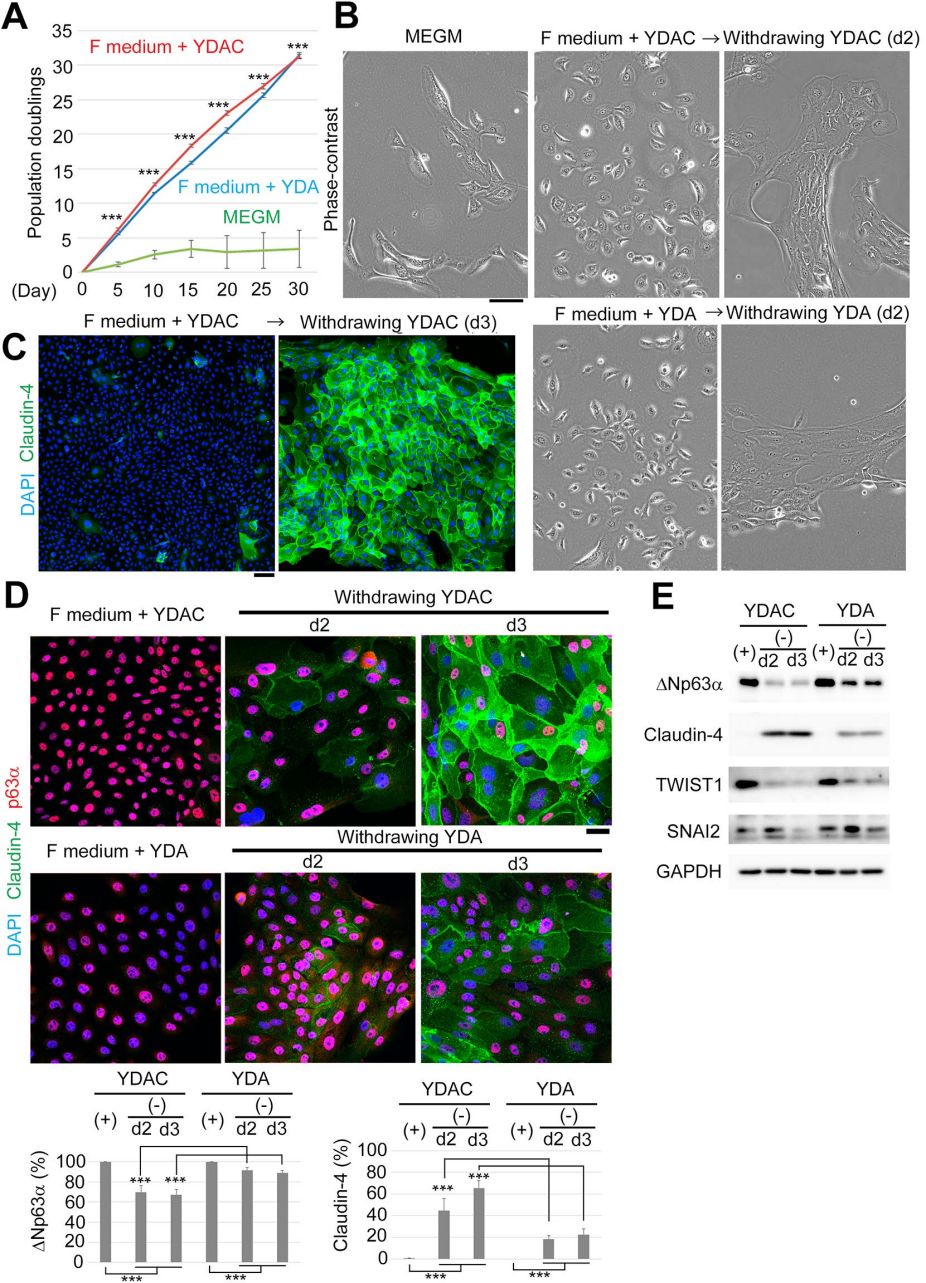
YDAC-containing F medium can better expand the mammary basal cell population that can express Claudin after drug withdrawal. **(A)** Plot of HMECs growth in each medium. Robust proliferation was observed in the F medium containing YDAC and YDA, but not in the commercial medium (MEGM). Three populations in each medium were counted every 5 days. **(B) **Phase-contrast micrograph showing withdrawal of YDAC and YDA results in an epithelium-like adhesive and flattened structure after 2 days (d2), which was not observed for cells kept in MEGM and inhibitor-containing F medium. **(C) **Immunostaining showing stronger Claudin-4 staining in the YDAC-removed cell population. Claudin-4 is a marker of tight junctions and epithelial differentiation. **(D) **Immunostaining comparing differentiation potential. Cells were maintained in YDAC- and YDA-containing F medium, and the compounds were withdrawn. Cells changed daily, becoming more Claudin-4-positive and less p63-positive. However, this change was more pronounced in YDAC-originated cells. Lower graph indicates the quantification of cells stained for each marker. Hundred cells were counted in each group (n=5). **(E)** Immunoblotting of samples in D showed similar results. Note YDAC-prominent changes in TWIST1 and SNAI2 after drug removal. Abbreviations of inhibitors: Y, 10 µM Y-27632; D, 1 µM DMH1; A, 1 µM A-83-01; and C,1 µM CHIR99021. Scale bars, 100 µm, 100 µm, and 10 µm in B, C, and D, respectively. All data were obtained from donor 1. See also Supplementary Figure 1 (donor 2, independent donors as biological replicates).

Thus, we first examined whether Wnt stimulation by CHIR99021 is important for the long-term expansion of primary HMECs (Fig. 1 and Supplementary Fig. 1). We prepared compound cocktails named YDAC (YDAC, 10 µM Y-27632, 1 µM DMH1, 1 µM A-83-01, and 1µM CHIR99021) and YDA (10 µM Y-27632, 1 µM DMH1, and 1 µM A-83-01). These were then added into F medium. Using them, we confirmed that both combinations could expand HMECs over 30 days more efficiently than MEGM, a commercially available primary culture medium (Fig. 1A and Supplementary Fig. 1A, biological replicates from independent donors).

We then examined how we can differentiate these expanding cells. We tried restoring ROCK and SMAD signaling by removing YDAC and YDA from the medium. Surprisingly, we found that these cells showed an epithelium-like adhesive and flattened structure in the phase contrast micrographs at 2 days after the removal, while the cells kept in MEGM and inhibitor-containing F medium did not (Fig. 1B). Immunostaining of the cells after YDAC removal confirmed robust expression of Claudin-4, a tight junction marker, suggesting that they were functionally differentiated into epithelium with high efficiency (Fig. 1C and Supplementary Fig. 1B, independent donors).

We further investigated whether YDAC or YDA provides greater differentiation potential to the Claudin-marked lineage. (Fig. 1D and E; Supplementary Fig. 1C and D; independent donors). Cell populations maintained with each cocktail were predominantly p63-positive, indicating that they primarily exhibited basal cell characteristics. After inhibitor removal, they became increasingly less p63-positive (decreasing to 70%) and Claudin-positive (reaching up to 70%). However, this change was more pronounced in YDAC-originated cells. In contrast, YDA-originated cells showed a decrease in p63 positivity to only 90% and Claudin positivity in up to only 30% of cells. We further extended the culture period of YDA-depleted cells to 5 days, but their differentiation state did not change significantly (Supplementary Fig. 1E, lower magnifications). Notably, p63 corresponds to ΔNp63α in a previous report^35^ and in this study (Supplementary Fig. 1F).

Since Wnt signal is known to activate EMT in which Claudin expression is suppressed^39^, we compared the extent of changes in EMT transcription factors after YDAC and YDA depletion. As a result, we found that YDAC-treated cells showed a greater reduction in TWIST1 and SNAI2/SLUG expression on day 3 (Fig. 1E and Supplementary Fig. 1D). Therefore, we concluded that YDAC-containing Wnt signal is important for enhancing EMT and MET after compound withdrawal. Thus, YDAC-containing F medium is more effective than YDA-containing medium in expanding the mammary basal cell population that can express Claudin after drug withdrawal.

### Comprehensive analysis of cells with and without YDAC

**Fig. 2:**
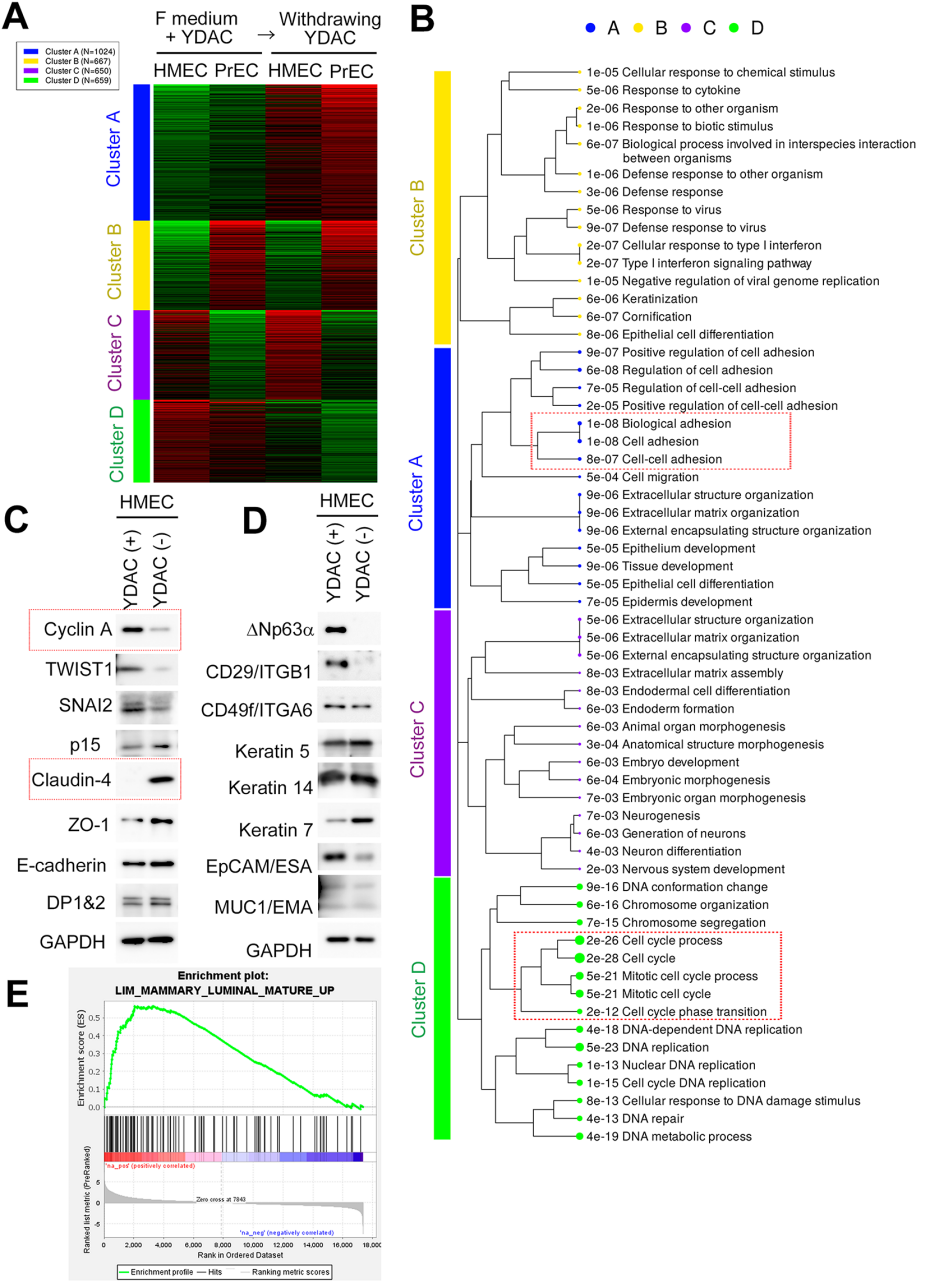
Comprehensive analysis of HMECs with and without YDAC. **(A) **K-means clustering dividing the top 3,000 genes into groups. K=4. Microarray data sets were derived from HMECs and PrECs before and after YDAC removal for 2 days (GSE254887 and GSE283321, respectively). K-means clustering was performed using log2-transformed and quantile-normalized microarray expression values. Each cluster represents genes exhibiting similar expression patterns across the experimental conditions. Actual values are provided in Supplementary Table 1. Heatmap colors indicate relative gene expression levels (red, high expression; blue, low expression). The data was obtained using iDEP96, an online analysis tool. See also Materials and Methods section. **(B) **Visualization of the top 15 enriched GO terms in each Cluster. Note the enrichment of GO terms related to cell cycle in cluster D and cell adhesion in cluster A. Actual gene sets are available in Supplementary Table 2. **(C, D)** Immunoblotting comparing marker expressions of HMECs before and after YDAC removal (YDAC (+) and YDAC (-), respectively). **(C)** Common cell biological markers: cell cycle markers, Cyclin A and p15; EMT markers, TWIST1 and SNAI2; AJC markers, Claudin-4, ZO-1, E-cadherin, and DP/Desmoplakin 1 & 2. **(D)** Mammary tissue-specific markers:Basal markers; p63, CD29/ITGB1, CD49f/ITGA6, Keratin 5, and Keratin 14; luminal markers, Keratin 7; Mamary grand markers, EpCAM/ESA and MUC1/EMA. Note that YDAC (+) cells show both basal cell properties and cell growth, while YDAC (-) cells show both epithelial differentiation properties and cell cycle arrest. The p63α antibody used recognizes ΔNp63α [35], Supplementary Figure 1 F).** (E)** GSEA-Preranked analysis on the log2 fold changes of gene expression derived from microarray differential expression analysis after YDAC-removal (GSE254887). The gene set (LIM_MAMMARY_LUMINAL_MATURE_UP) was significantly enriched (NES = 1.98, p = 0.0, FDR q-value = 0.0). The core enriched genes (leading edge) included TGM2, SORT1, FOXA1, WNT7B, and PTPN6, all previously reported as highly expressed in mature luminal mammary cells [40]. The enrichment plot clearly indicated significant enrichment among top-ranked genes. Analysis was conducted using GSEA software (version 4.3.2). The core enriched genes identified by GSEA-Preranked analysis are listed in Supplementary Table 3.

We then comprehensively analyzed changes in gene expressions in cells derived from two types of p63-positive bilayered epithelia—the mammary and prostate glands—before and after YDAC-removal (Fig. 2 and Supplementary Tables 1–3). Microarray data was obtained from YDAC-treated and -removed HMECs or PrECs (prostate epithelial cells) and compared. Gene clustering by k-means revealed that the gene expression can be clustered into 4 clusters. Especially, cluster A and D showed YDAC removal-dominant genes and YDAC treatment-dominant ones, respectively (Fig. 2A and Supplementary Table 1). Following GO analysis focusing on biological process revealed that genes enriched in cluster D show cell cycle and mitosis related GO terms, suggesting cell proliferation (Fig. 2B and Supplementary Table 2). Additionally, genes enriched in cluster A show cell adhesion-related GO terms including Claudins, suggesting epithelial differentiation. Thus, we estimated that YDAC treatment maintains basal cells in a proliferative and undifferentiated state, and YDAC removal makes them exit the cell cycle and differentiate.

We then experimentally confirmed it in HMECs by investigating the changes in the amount of marker proteins before and after YDAC removal (Fig. 2C and D). First, we used the common cell biological markers including proliferation, epithelial differentiation, and EMT transcriptional markers (Fig. 2C). Among them, cells maintained in YDAC predominantly expressed cyclin A, a cell proliferation marker, and TWIST1 and SNAI2, EMT transcription factors. Following YDAC removal, the cells lost expressions of cyclin A and the EMT transcription factors. They also upregulated CDK inhibitor p15INK4B/CDKN2B expression, suggesting they exited the cell cycle due to differentiation. Indeed, we found an increase especially in Claudin-4 and ZO-1 after YDAC removal, which are functional tight junction proteins at apical junction complex (AJC) reflecting epithelial differentiation. In contrast, the increases in DP/Desmoplakin and E-cadherin were not significant.

Next, we examined mammary tissue-specific markers, including basal and luminal ones (Fig. 2D). We found that YDAC-maintained cells predominantly expressed basal cell markers of p63 and CD29/ITGB1, which were clearly diminished after YDAC removal. YDAC-maintained cells also expressed other basal cell markers of CD49f/ITGA6: Keratin 5, Keratin 14. However, they didn’t diminish after YDAC removal. YDAC-removed cells showed prominent expression of Keratin 7, a luminal marker. Mammary gland markers of EpCAM/ESA, and MUC1/EMA were observed in both conditions.

Finally, we evaluated how YDAC-removed mammary cells differentiate toward a mature mammary luminal lineage. We calculated log2 fold changes in gene expression following YDAC removal and performed GSEA-Preranked analysis using a previously published gene signature of mature mammary luminal cells (LIM_MAMMARY_LUMINAL_MATURE_UP)^40^, utilizing the GSEA software (Figure. 2E and Supplementary Table 3)^41,42^. The analysis demonstrated significant enrichment of this gene set in YDAC-removed cells (NES = 1.98; *p* = 0.0, FDR (q-value) = 0.0). Core enriched genes included TGM2, SORT1, FOXA1, WNT7B, and PTPN6, all known markers highly expressed in mature luminal cells, functioning as a cytoplasmic protein, a cytoplasmic protein, a transcription factor, a signaling ligand, and a cytoplasmic protein, respectively^40^. In contrast, marker genes such as MYB, ESR1 (estrogen receptor), ALCAM, BTRC, and GADD45G were not identified as core enrichment, that might result from the limited scale of our dataset. Nonetheless, the clear enrichment of the mature luminal gene signature strongly indicates that differentiation in YDAC-removed mammary cells extends beyond Claudin expression alone.

Overall, these marker profiles indicate that YDAC can expand mammary basal cells before differentiation, and YDAC removal results in Claudin-prominent epithelial differentiation and cell cycle exit. Though basal markers show some redundancy in YDAC-removed cells, the expression of Claudin, a functional marker of tight junction, the enrichment of gene sets in mammary luminal cells, and cell cycle exit is evident in YDAC-removed cells. Hence, cells expressing Claudin by YDAC removal are hereafter referred to as “differentiated cells.” Additionally, cells suppressing Claudin expression by YDAC treatment are referred to as “undifferentiated cells.”

### EGR1 and ELF3 are mutually exclusive markers for undifferentiated basal cells and Claudin-expressing epithelia, respectively

Our 2D culture method does not require traditional feeder cells and allows effective differentiation of mammary basal cells by simply removing the inhibitor cocktail. These advantages allow the clear separation of undifferentiated basal cell populations and differentiated ones, with high purity.

**Fig. 3:**
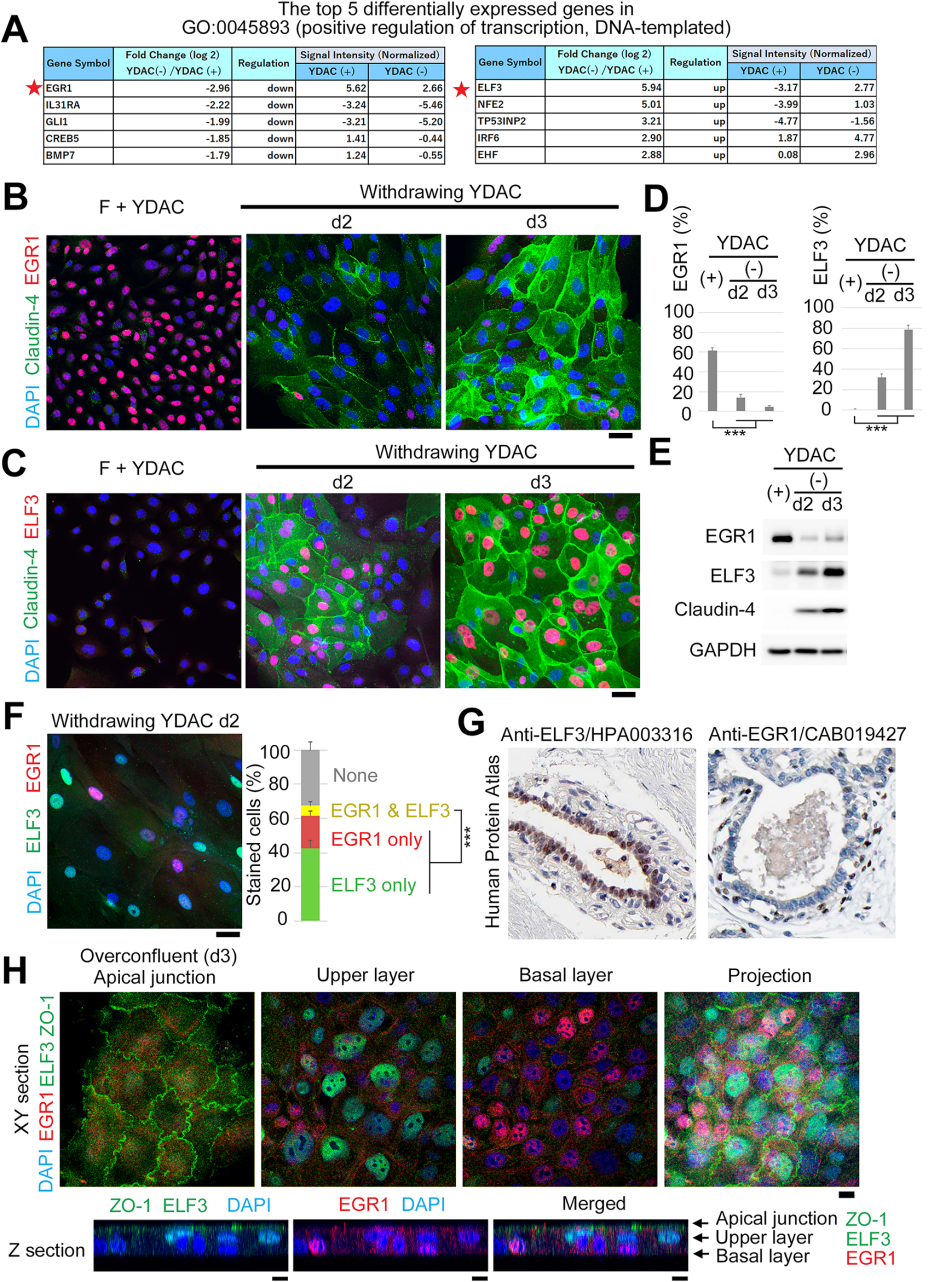
EGR1 and ELF3 are mutually exclusive markers for undifferentiated and differentiated HMEC cells, respectively. **(A) **Top five differentially expressed genes in GO term: 0045893 (positive regulation of transcription, DNA-templated). Microarray data sets were compared between cells before and after YDAC removal for 2 days (YDAC (+) and YDAC (-), respectively).** (B, C) **Immunostaining showing EGR1 expression in nearly half of the YDAC-maintained undifferentiated basal cell population. EGR1 expression was rare after differentiation through YDAC removal. In contrast, ELF3 expression was rare in YDAC-maintained cells, but observed in nearly half of all cells 3 days after YDAC removal. Note that cells with a decrease in EGR1 and an increase in ELF3 almost coincided with those expressing Claudin-4. **(D) **Quantifications of stained cells and **(E) **immunoblotting with samples in B and C supports the data. Hundred cells were counted in each group (n=5). **(F) **Left, Double staining for EGR1 and ELF3 during the transient status (day 2). Note the mutually exclusive expression. Right, the quantification. **(G) **Tissue expression data from the Human Protein Atlas (HPA) website, where anti-ELF3 stains differentiated luminal cells, and anti-EGR1 partially stains basal cells, including undifferentiated cells. The specificity of antibodies is validated by the presence of single bands in endogenous immunoblotting (see also Materials and Methods section). **(H) **Our new method for mammary cell culture in an in vivo-like bilayer. The bilayer was created by culturing cells in YDAC medium for approximately 3 days after they reached confluence (see also Supplementary Figure 2 G). Immunostaining and confocal microscopy analysis revealed ZO-1 and ELF3 expressions in the upper layer and EGR1 expression in the lower layer. Note their relevance with the natural organization of mammary glands in G and mammary organoids in Supplementary Figure 2 F. Scale bars, 10 µm. All cell data were obtained from donor 1. See also Supplementary Figure 2 (donor 2, independent donor as a biological replicate).

Utilizing this merit, we sought to identify key transcriptional factors that determine Claudin expression in HMECs (Fig. 3 and Supplementary Fig. 2). We compared the microarray data before and after YDAC removal and extracted differentially expressed genes (DEGs), especially in the GO term of transcriptional regulation (Fig. 3A). As a result, two transcription factors of EGR1 and ELF3 were identified as the greatest DEGs in the fold-change before and after differentiation, respectively. EGR1 reportedly functions in stress response and cancer progression^43,44^, and ELF3 functions in the development of the intestine and the lung^45^. However, both functions in healthy tissue, especially in the mammary epithelium, are to be determined.

We examined EGR1 and ELF3 in our cellular systems. Through immunostaining and immunoblotting, we confirmed the expression of these proteins before and after differentiation (Fig. 3, B-E; and Supplementary Fig. 2, A-D; independent donors). Our findings showed that EGR1 was expressed in nearly half of the basal cells kept in YDAC, but was hardly expressed following YDAC removal. Meanwhile, ELF3 was hardly expressed in the YDAC-maintained cells, with expression noted in nearly half of them after differentiation. Furthermore, we observed that cells showing downregulation of EGR1 and upregulation of ELF3 overlapped with those expressing Claudin-4 (Fig. 3B and C, Supplementary Fig. 2A and B; independent donors). Indeed, double staining for EGR1 and ELF3 during the transient status (day 2) revealed that their endogenous expression is mutually exclusive (Fig. 3F and Supplementary Fig. 2E). Thus, the expression patterns of EGR1 and ELF3 are distinct and more clearly distinguishable than that of p63. Therefore, we speculated that EGR1 and ELF3 could serve as more sensitive markers for undifferentiated and differentiated cells regarding Claudin expression, respectively.

Next, we attempted to confirm these findings in tissue samples. First, our observations are supported by tissue expression data from the Human Protein Atlas (HPA), which were obtained using validated antibodies. As shown in Fig. 3G, anti-ELF3 staining clearly distinguishes luminal cells, while anti-EGR1 staining partially marks basal cells, which is consistent with some of them possessing differentiation potential (original data; anti-ELF3/HPA003316, https://www.proteinatlas.org/ENSG00000163435-ELF3/tissue/breast; anti-EGR1/CAB019427, https://www.proteinatlas.org/ENSG00000120738-EGR1/tissue/breast). The specificity of both antibodies has been validated by the developers, as demonstrated by the presence of single bands in endogenous immunoblotting.

We further developed a simple culture method for HMECs to assemble into in vivo-like bilayers (Fig. 3H and Supplementary Fig. 3G). The bilayer was established by culturing cells in YDAC-containing medium for approximately 3 days after reaching confluence (Supplementary Fig. 3G). During this period, overgrown cells become compressed and protruded into the upper layer, a process that may mimic forced cell rounding, which is known to induce epithelial differentiation^37^, potentially counteracting the effect of YDAC. Through immunostaining and confocal microscopy, we found that ZO-1 and ELF3 were expressed in the upper layer, while EGR1 was expressed in the lower layer (Fig. 3H). This arrangement is consistent with the natural organization of mammary bilayers, where the upper layer consists of luminal epithelial cells and the lower layer consists of basal cells including cells with differentiation potential (Fig. 3G).

Finally, we introduced organoids derived from our culture and examined the relevance of our findings (Supplementary Fig. 2F). HMECs maintained in YDAC formed well-polarized cystic structures in Matrigel, as indicated by phalloidin staining. Moreover, EGR1-marked cells tended to be observed at the periphery, suggesting a basal localization. In contrast, ELF3-marked cells were observed on the inner side, suggesting localization within the inner layer. Furthermore, these findings suggest that the organoids exhibit heterogeneity in differentiation, as indicated by the partial expression of EGR1 and ELF3.

Thus, the distributions of EGR1 and ELF3 observed in our 2D models are recapitulated in bilayer models, mammary organoids, and tissues, further supporting their utility as differentiation state-specific markers.

### Endogenous EGR1 suppresses Claudin expression

**Fig. 4:**
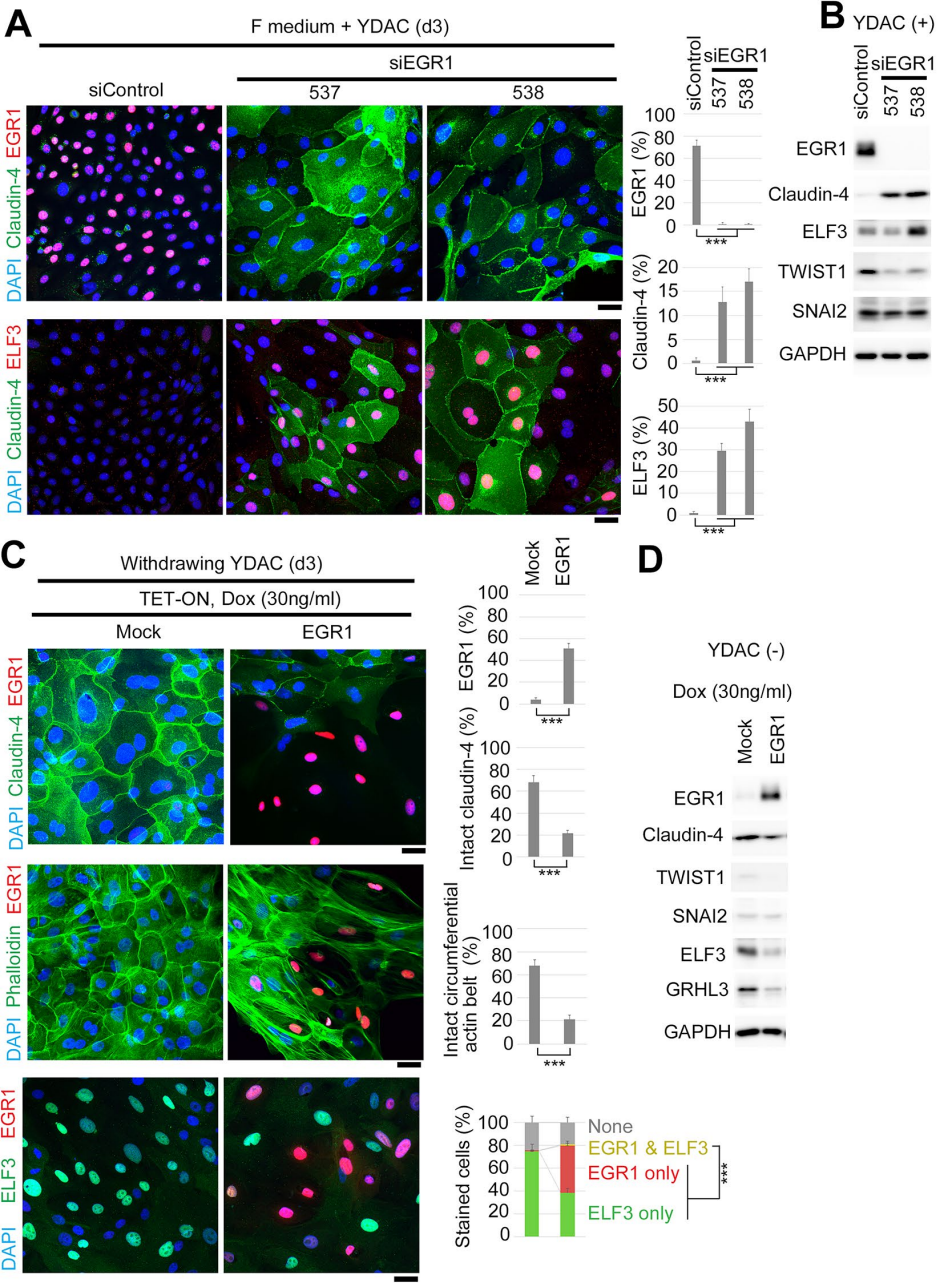
Endogenous EGR1 suppresses Claudin expression in HMECs.**(A)** Left, immunostaining of YDAC-maintained undifferentiated basal cell population treated with siEGR1 for 3 days, which caused the disappearance of EGR1 expression and an increase in the population of Claudin-4- and ELF3-stained cells. 537 and 538 are the designations of siRNAs. Right, quantification of cells stained with each marker. Hundred cells were counted in each group (*n* = 5). **(B)** Immunoblotting with samples in A supports the data. **(C)** Ectopic induction of EGR1 in differentiated cells with Dox-containing YDAC-free F medium. Left, immunostaining indicating disappearance of Claudin-4 at cell-cell borders (top), impaired formation of actin circumferential ring (middle), disappearance of ELF3 (bottom), in ectopically EGR1-expressing cells. Right, quantification of cells stained with each marker. Hundred cells were counted in each group (*n* = 5). **(D)** Immunoblotting with samples in C. Mock, empty vector. siControl corresponds to a negative control. Scale bars, 10 μm.

Then, we analyzed the function of EGR1 in our HMEC culture model (Fig. 4 and Supplementary Fig. 3, independent donors). We treated YDAC-maintained undifferentiated cells with siEGR1. Surprisingly, EGR1 knocked down cells exhibited differentiation, as indicated by an increase in Claudin-4 in both immunostaining and immunoblotting (Fig. 4A and B; Supplementary Fig. 3A and B). This is consistent with organoids treated with siEGR1, which formed smaller aggregates, suggesting growth arrest due to early differentiation (Supplementary Fig. 3C).　ELF3-stained cells were also increased (Fig. 4A and B; Supplementary Fig. 3A and B), suggesting that EGR1 takes part in suppressing expressions of these proteins. As EGR1 in cancer cell lines promotes the transcription of EMT-related transcription factors^44,46^, we examined the amount of TWIST1 and SNAI2 and found that TWIST1 is downregulated by EGR1 knockdown(Fig. 4B; Supplementary Fig. 3B). TWIST1 expression has been reported to correlate with the downregulation of Claudin-4 in esophageal cancer tissues and in cell lines overexpressing TWIST1^47^. Thus, we concluded that EGR1 in the basal cell maintains TWIST1 expression, that is important for suppression of Claudin expression.

Next, we examined the ectopic effect of EGR1 by aberrantly expressing them in YDAC-withdrawn differentiated cells (Fig. 4C and D). We established a pooled population of HMEC cells inducibly expressing EGR1 under doxycycline (Dox) treatment (TET-ON HMEC system, see also Materials and Methods section). Then, we treated them with Dox under YDAC-depleted condition and found that ectopically expressed EGR1 can suppress Claudin localization at the cell-cell border (Fig. 4C, top). However, the amount of TWIST1 and SNAI2 are not elevated (Fig. 4D), suggesting EGR1 does not promote EMT in YDAC-removed condition. Moreover, the decrease in the protein level of Claudin-4 is small (Fig. 4D). Notably, the formation of a circumferential actin belt highlighted by phalloidin staining is disturbed (Fig. 4C, middle), As the circumferential actin belt is important for the formation of tight junctions^2^, this result suggests that epithelial cell polarity is incomplete. Moreover, exogenous EGR1 exclusively decreased ELF3 expression in both immunostaining and immunoblotting (Fig. 4C, bottom; and D). Together with mutually exclusive expressions of EGR1 and ELF3 in endogenous and knockdown conditions (Figs. 3 and 4A, Supplementary Fig. 2, Supplementary Fig. 3A), we speculated that the disturbance of ELF3 expression might correlate with actin localization and could explain the EGR1 effect on Claudin localization.

### Endogenous ELF3 localizes Claudin at cell-cell border

**Fig. 5:**
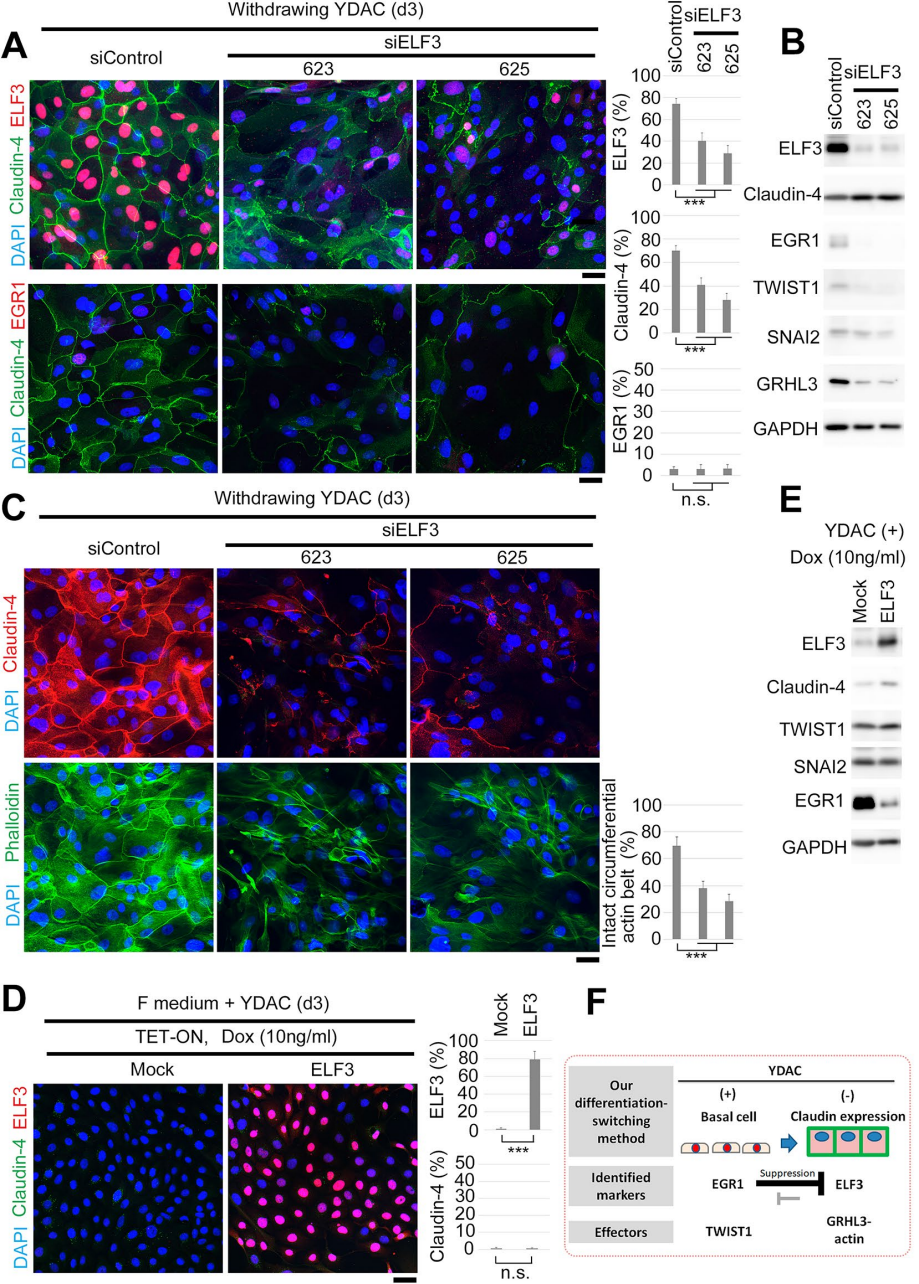
Endogenous ELF3 localizes Claudin at cell-cell border in HMECs.**(A)** Left, immunostaining of YDAC-maintained basal cell population treated with siELF3, whereafter YDAC was withdrawn for 3 days to induce differentiation. ELF3 knockdown cells showed a disturbance in Claudin-4 localization at cell-cell borders. Note that, 623 and 625 are the designations of siRNAs. Right, quantification of cells stained with each marker. Hundred cells were counted in each group (*n* = 5). **(B)** However, immunoblotting did not confirm ether decrease in Claudin-4, or increase in EGR1, TWIST1, and SNAI2 but confirmed a decrease in actin organizer GRHL3, implying the importance of ELF3 not for EMT but for cell polarity in the differentiated state. **(C)** Left, another immunostaining with the same sample in A, indicating impaired formation of actin circumferential ring in ELF3 knockdown cells, with similarity to ectopic EGR1 expression. Right, quantification of cells. Hundred cells were counted in each group (*n* = 5). **(D)** Ectopic induction of ELF3 in undifferentiated basal cells with Dox-and YDAC-containing F medium. Left, immunostaining indicating no increase in Claudin-4 staining in ectopically ELF3-expressing cells. Right, quantification of cells stained with each marker. One-hundred cells were counted in each group (*n* = 5). **(E)** Immunoblotting with samples in D. **(F)** Graphical abstract of our cellular model and findings. See also: main text. Mock, empty vector. siControl corresponds to a negative control. Scale bars, 10 μm.

Then, we analyzed the function of endogenous ELF3 in differentiated HMEC cells (Fig. 5 and Supplementary Fig. 4, independent donors). We initially treated YDAC-maintained cells with siELF3 and then withdrew YDAC to differentiate them. Notably, ELF3 knocked down differentiated cells showed a disturbance in Claudin-4 localization at cell-cell borders (Fig. 5A and Supplementary Fig. 4A). However, immunoblotting did not confirm either a decrease in the amount of Claudin-4 or increases in EGR1, TWIST1, and SNAI2, suggesting that the results are not due to EMT (Fig. 5B and Supplementary Fig. 4B).

We focused on epithelial cell polarity and stained the cells with phalloidin as in the case of EGR1 overexpression. Notably, we found that ELF3 knocked down cells disrupted Claudin localization and formed incomplete circumferential actin belts (Fig. 5C and Supplementary Fig. 4C). This is consistent with organoids treated with siELF3, which developed a bumpy shape, suggesting disordered polarity due to an incomplete actin network (Supplementary Fig. 4D). In addition, we found that GRHL3 is also diminished in immunoblotting of ELF3 knockdown cells (Fig. 5B and Supplementary Fig. 4B). GHRL3 is a target gene of ELF3 during MET^48^ and is independently known as an actin network organizer during development and epidermal differentiation^49^. Thus, we concluded that ELF3 in differentiated HMECs maintains GRHL3 expression, which is important for the formation of a circumferential actin belt, thereby localizing Claudin at cell-cell borders. This mechanism can explain the phenotype of EGR1 overexpression exclusively downregulating ELF3 and the downstream GRHL3-actin organization axis (Fig. 4C and D).

Finally, we examined the ectopic effect of ELF3 by aberrantly expressing them in YDAC-maintained undifferentiated cells (Fig. 5D and E). We established a pooled population of ELF3-inducible HMEC cells (TET-ON HMEC system) and induced them by doxycycline under YDAC-existing conditions. Then, we found that ectopically expressed ELF3 could decrease the level of EGR1 in immunoblotting but not adequately elevate the Claudin level both in localization and immunoblotting. Additionally, decreases in the level of TWIST1 and SNAI2 were not observed. Overall, we conclude that the ectopic expression of ELF3 in undifferentiated basal cells can downregulate the amount of EGR1 to some extent, suggesting mutual exclusion. However, it is not sufficient to impact EMT suppression, suggesting the complexity and robustness of EMT regulation in normal cells^39^.

In conclusion, we confirmed that EGR1 and ELF3 are mutually exclusive and act as functional markers of undifferentiated and differentiated HMECs, respectively (Fig. 5F). These findings highlight the utility of our culture method for discovering differentiation state-specific markers and their functional validation regarding Claudin expression.

## Discussion

Here, we introduced two normal cell culture models: (1) the long-term expansion of mammary basal cells, including undifferentiated ones, using YDAC-containing F medium, and (2) a rapid differentiation method for Claudin expression by YDAC removal (Fig. 1, Supplementary Fig. 1). Using these models, we combined comparative microarray analysis and cellular assays, which identified EGR1 and ELF3 as markers of undifferentiated and differentiated states, respectively (Figs. 3, 4 and 5, Supplementary Figs. 2–4). The mechanisms likely involve TWIST1-mediated epithelial-mesenchymal transition (EMT) and GRHL3-mediated actin organization, respectively. Also, their relevance was observed in tissues and organoids. Thus, our system provides a simplified model for epithelial differentiation in terms of Claudin regulation (Fig. 5F).

Our cell culture system has several technical advantages. It isolates pure cell populations in distinct differentiation states, overcoming tissue heterogeneity that can obscure key markers. Unlike traditional immortalized epithelial cell lines such as EPH4 and Caco-2, which remain in a constantly differentiated state, or cell lines with altered TGF-β signaling, such as NMuMG and A549^48,50^, our naïve primary cells enable functional analyses of normal differentiation pathways. Additionally, our system overcomes limitations of conventional gene-targeted animals, which often face challenges such as functional redundancy and difficulty in multiple interventions. Thus, using our simplified culture system, we could highlight the significance of EGR1 and ELF3 in normal basal cell differentiation (Fig. 3 and Supplementary Fig. 2).

Our system identified the function of EGR1 in normal mammary differentiation and suggested its underlying mechanism (Fig. 4, Supplementary Fig. 3). In tissues, EGR1 is a transcription factor that is rapidly induced by cytokines in response to tissue damage^43,44^. Comprehensive studies on mammary tissues have observationally shown that EGR1 expression is enhanced in basal cells^22,51^. While EGR1-knockout models show no overt defects in early development, they exhibit a lack of epithelial ductal elongation and dichotomous branching in adulthood, which is presumed to result from ovarian hormonal imbalances^52^. In contrast to these observational studies, our model clearly demonstrated that the EGR1-TWIST1 axis is crucial for maintaining undifferentiated basal cells. This finding is also consistent with these in vivo studies showing its role in tissue damage response and mammary gland maintenance. EGR1 has also been implicated in cancer cell growth, EMT, and metastasis^44,46,53^. We further found that, unlike in cancer cells where EGR1 promotes SNAI1/SNAIL expression^44,46^, its downstream in normal mammary cells appears to be distinct.

We also identified a novel role of ELF3 in normal epithelial differentiation (Fig. 5, Supplementary Fig. 4). ELF3 (ESE-1) is a transcription factor belonging to the ETS family^54^, known for its role in epithelial homeostasis and differentiation^45^. However, its function in mammary differentiation remains unclear, possibly due to molecular compensation in knockout studies. This contrasts with ELF5, which is well-studied in this context^21,55–57^. Our model clearly linked ELF3 to GRHL3-mediated actin organization and Claudin localization (Fig. 5, Supplementary Fig. 4), that is consistent with in vivo studies showing its role in tissue homeostasis and differentiation. The results also suggest a normal mammary grand-specific ELF3 axis distinct from MET regulation in others, because ELF3’s downstream molecules vary across cell types^48^. Notably, ELF3 loss in bile ducts, commonly observed in cancers, leads to ZEB2 and EMT-related protein upregulation, reducing cell-cell junction proteins and increasing cell motility^58^. However, this cancer-associated role of ELF3 is distinct from our findings in normal mammary epithelia, where Claudin level remains unchanged.

Our findings on EGR1 and ELF3 modulation highlight the value of our system in studying partial EMT, an intermediate state where cells exhibit both epithelial and mesenchymal characteristics. Partial EMT has been implicated in cancer metastasis, migration, and chemotherapy resistance due to its plasticity and adhesive collective migration^3,39,59^. Our data suggest a mixed EMT phenotype, characterized by (1) co-expression of Claudin-4 and SNAI2 upon EGR1 knockdown (Fig. 4B, Supplementary Fig. 3B) and (2) a hybrid epithelial-mesenchymal state, where Claudin expression is maintained, but epithelial polarity is disrupted as a result of ELF3 knockdown and EGR1 overexpression (Fig. 5A-C, Supplementary Fig. 4). Claudin has been shown to contribute to partial EMT in vivo, promoting cancer progression. Specifically, Claudin-1 expression has been associated with: (1) co-expression of SNAI2 and ZEB1 in hepatocellular carcinoma^60^, (2) EMT via the AMPK/TGF-β pathway in head and neck cancer^61^, and (3) cancer cell migration through partial EMT mechanisms^62,63^. In these contexts, Claudin-mediated partial EMT is crucial for tumor invasion and metastasis. While our current data do not directly link to cancer progression, further intervention will enhance our system’s value for studying partial EMT in cancers, in terms of Claudin expression.

Although our primary cells retain in vivo properties, culture conditions may introduce artifacts. Some basal and luminal markers showed redundant expression, suggesting incomplete niche recapitulation (Fig. 2D). To validate our data, we performed GSEA using mammary lineage signatures, analyzed tissue localization using public databases, and employed organoids and a new bilayer culture system (Figs. 2E and 3F, and 3G, Supplementary Figs. 2 F, 3 C, and 4D), which strengthened the consistency of our results.

In summary, our naïve basal cell model facilitates the identification of key differentiation markers related to Claudin expression, bridging conventional cellular models and in vivo tissues. This system holds promise for applications in epithelial physiology, pharmacology, regenerative medicine, and cancer research. Future directions include testing differentiation-inducing factors, optimizing culture methods to establish biologically functional cells, and developing cancer models for drug discovery. Ongoing research in our lab continues to explore these applications.

## Materials and methods

### Cell culture

HMECs and PrECs were purchased from Lonza (Basel, Switzerland). Long-term expansion of basal cells without feeder cells was achieved using F medium^33^ and our four-inhibitor cocktail, termed YDAC. F medium was composed of 3:1 (v/v) Ham’s F-12 (087–08335, Wako, Tokyo, Japan) and DMEM (043–30085, Wako), 5% fetal bovine serum, 0.4 µg/mL hydrocortisone (086–10191, Wako), 5 µg/mL insulin (I9278, Sigma, St. Louis, MO, USA), 8.4 ng/mL cholera toxin (030–20621, Wako), 10 ng/mL epidermal growth factor (059–07873, Wako), 24 µg/mL adenine (A2786, Wako), 100 µg/mL streptomycin (Meiji Seika, Tokyo, Japan), 100 U/mL penicillin (Meiji Seika), 250 ng/mL Amphotericin B (A2942, Sigma), and 10 µg/mL gentamicin (078–06061, Wako). YDAC is a combination of 10 µM Y-27632 (1000583, Cayman Chemical, Ann Arbor, MI, USA), 1 µM DMH1 (041–33881, Wako), 1 µM A-83-01 (039–24111, Wako), and 1 µM CHIR99021 (038–23101, Wako). HMECs were also grown in a commercially available medium (MEGM Mammary Epithelial Cell Growth Medium BulletKit, CC-3150, Lonza).

Differentiation in 2D culture was achieved by cultivating the basal cells in F medium without inhibitors for 2–3 days. Differentiation into bilayered mammary epithelium was achieved by continuously cultivating the basal cells in YDAC-containing F medium for 2–3 days after confluence.

Population doubling was calculated as follows: *n* = 3.32 (log UCY - log l) + X, where n = the final PDL number at the end of a given subculture, UCY = the cell yield at that point, l = the cell number used as inoculum to begin that subculture, and X = the doubling level of the inoculum used to initiate the subculture being quantitated.

All cells were maintained at 37 °C in a humidified incubator with 5% CO_2_. Donor 2 was used for biological duplicates.

### Organoid culture, fixation, immunostaining, and functional siRNA assay

HMECs maintained in YDAC-containing medium were used. For organoid culture, single-cell suspensions of HMECs were embedded in growth factor-reduced Matrigel (354230, Corning, NY, USA) and cultured under defined conditions. Briefly, 30 µL of Matrigel was placed into each well of a pre-warmed 24-well plate and allowed to polymerize at 37 °C for 15–30 min. Subsequently, 30 µL of Matrigel containing HMECs at a density of 1 × 10⁴ cells/mL was overlaid onto the solidified Matrigel. YDAC-containing F medium was used as the culture medium and was changed every 3–4 days for up to 8 days. In the subsequent experiments, the experimental equipment was pre-coated with blocking buffer (10% FBS in phosphate-buffered saline (PBS)).

For fixation, Matrigel was dissolved in Cell Recovery Solution (354253, Corning) for 1 h at 4 °C. The organoids were then centrifuged at 70 × g for 5 min, the supernatant was removed, and the organoids were fixed with 1% formaldehyde for 1 h at room temperature. After fixation, the organoids were permeabilized with 0.5% Triton X-100 in PBS for 30 min. Blocking was performed for at least 1 h at room temperature.

For immunostaining, organoids were incubated with primary antibodies in Canget Signal Solution A (NKB-501, TOYOBO, Osaka, Japan) overnight at 4 °C, washed three times with blocking solution, and then incubated with secondary antibodies in Canget Signal Solution A overnight at 4 °C. Nuclei were counterstained with DAPI, and filamentous actin was visualized using Alexa 488-conjugated Phalloidin (A12379, Thermo Fisher Scientific, Waltham, MA, USA).

For the functional assay, siRNAs validated in our study were introduced into organoids on day 4 using a published protocol^64^. In brief, siRNA complexes were formed using RNAiMAX (Thermo Fisher Scientific) in YDAC-containing F medium, which included FBS to improve transfection efficiency. Samples were harvested after 4 days.

### Antibodies, siRNAs, and compounds

The following primary antibodies were used: rabbit anti-p63α (Cat: sc-8344, Santa Cruz, Dallas, TX, USA), mouse anti-Claudin-4 (32–9400, Zymed, San Francisco, CA, USA), rabbit anti-TWIST1 (25465-1-AP, Proteintech, Rosemont, IL, USA), rabbit anti-SNAIL/SNAI1 + SLUG/SNAI2 (ab85936, abcam, Cambridge, UK ), mouse anti-Cyclin A (611268, BD, Franklin Lakes, NJ, USA), rabbit anti-INK4b/p15 (sc-613, Santa Cruz), mouse anti-ZO-1 (610967, BD), rat anti-E-cadherin (ECCD2, TaKaRa, Shiga, Japan), guinea pig anti-Desmoplakin 1/2 (DP-1, PROGEN, Heidelberg, Germany), rabbit anti-CD29/ITGB1 (26918-1-AP, Proteintech), rabbit anti-CD49f/ITGA6 (EPR5578, abcam), guinea pig anti-Keratin 5 (BP5006, Acris, Herford, Germany), rabbit anti-Keratin 14 (10143-1-AP, Proteintech), mouse anti-Keratin 7 (66483-1-Ig, Proteintech), rabbit anti-EpCAM/ESA (21050-1-AP, Proteintech), rabbit anti-MUC1/EMA C-terminal (23614-1-AP, Proteintech), rabbit anti-EGR1 (#4154, Cell Signaling Technology, Massachusetts, USA), rabbit anti-ELF3 (HPA003479, Sigma), mouse anti-GRHL3 (sc-398838, Santa Cruz), and mouse anti-GAPDH (sc-32233, Santa Cruz). DAPI was purchased from Dojindo (Kumamoto, Japan).

Silencer Select siRNAs (Thermo Fisher Scientific) were used for human gene knockdown. siRNA IDs were as follows: EGR1, s4537, and s4538; ELF3, s4623, and s4625. Silencer Select siRNA control no. 1 (Thermo Fisher Scientific) was used as the negative control. Cells were transfected with Lipofectamine RNAiMAX (Life Technologies, Carlsbad, CA, USA). siRNA-transfected basal cells were grown in YDAC-containing F medium for 3 days. To incorporate the differentiation process, transfection was performed in YDAC-containing F medium for 4 h, prior to 3 days of cultivation in F medium without inhibitors.

### TET-ON HMEC system

A pooled population of TET-ON HMECs, in which gene expression is induced by doxycycline (Dox; TaKaRa), was produced by lentiviral infection as previously described^65–67^. The concentrations and treatment durations of Dox are indicated in figures.

### Genes

GATEWAY clones with cDNAs encoding full-length human EGR1 and ELF3 were purchased from DNASU^68^ (HsCD00296279 and HsCD00000232, respectively; DNASU, AZ, USA), and introduced into TET-ON HMEC system.

### Cell staining

Cells were grown on coverslips (Iwaki Glass Co., Ltd., Tokyo, Japan). Formaldehyde fixation was performed as previously described^28^. Briefly, cells were fixed with 1% formaldehyde in PBS for 15 min, blocked in 1% bovine serum albumin/PBS for > 15 min, and incubated for 1 h with primary antibodies. After washing three times in PBS, cells were incubated for 30 min with secondary antibodies. In some experiments, primary antibodies were conjugated to fluorescent dyes using the Zenon Labeling Kit (Thermo Fisher Scientific).

### Image acquisition and analysis

Phase-contrast images were obtained by using the BZ-X800 system (KEYENCE, Tokyo, Japan), equipped with a Plan Apochromat 20×/0.75 NA lens (KEYENCE), a Peltier cooled CCD camera (KEYENCE), and BZ-X800 acquisition software (KEYENCE). Micrographs were captured by using an FV3000 confocal laser-scanning microscope equipped with a UPlanSApo 40×/0.95 NA and 20x/0.75 lens and the FV31S-SW as the imaging software (all from Olympus, Tokyo, Japan). All images were further processed using Photoshop Elements 2018, according to *Scientific Reports’* guidelines.

### Gene expression microarray and data analysis

Gene expression microarray analyses were performed using the SurePrint G3 Human GE Microarray 8 × 60 K Ver3.0 (Agilent Technologies, Palo Alto, CA, USA) with support from DNA Chip Research Inc. (Tokyo, Japan). Probes flagged as Detected were selectively retained. The data have been deposited in the Gene Expression Omnibus (GEO) database under accession no. GSE254887 (HMECs) and GSE283321 (PrECs). Microarray expression values were log2-transformed and quantile-normalized.

Further analyses, including k-means clustering and GO analyses, were performed using the iDEP.96 online tool (http://bioinformatics.sdstate.edu/idep96/) with default parameters recommended by the developer^69^. The optimal cluster number (K) was determined by iDEP.96 based on the within-cluster sum of squares. Gene Ontology (GO) enrichment analyses were also conducted using the default settings of iDEP.96.

We performed GSEA-Preranked analysis on the log2 fold changes of gene expression derived from microarray differential expression analysis after YDAC-removal (GSE254887). The gene set LIM_MAMMARY_LUMINAL_MATURE_UP^40^　(https://www.gsea-msigdb.org/gsea/msigdb/human/geneset/LIM_MAMMARY_LUMINAL_MATURE_UP.html, *n* = 113 genes) was significantly enriched (NES = 1.98, *p* = 0.0, FDR q-value = 0.0). The core enriched genes were provided in Supplementary Table 3. The enrichment plot was provided in Fig. 2E. Analysis was conducted using GSEA software (version 4.3.3), using the default settings.

### Confirmation of protein localization in vivo.

The Human Protein Atlas (HPA) online database (http://www.proteinatlas.org) was used to retrieve immunohistochemistry images of the selected proteins^70^. All antibodies used were experimentally validated by the presence of single bands in endogenous immunoblotting as documented on the following websites. These include: anti-ELF3/HPA003316 (https://www.proteinatlas.org/ENSG00000163435-ELF3/summary/antibody) and (https://www.sigmaaldrich.com/AM/en/product/sigma/hpa003316); anti-EGR1/CAB019427/Cell Signaling #4153 (https://www.cellsignal.com/products/primary-antibodies/egr1-15f7-rabbit-mab/4153). URLs that link to the images used are provided in the main text.

### Statistical analysis

Data are presented as the mean ± standard deviation (SD). Statistical significance was determined using the two-tailed t-test in Excel Software (Microsoft, Redmond, WA, USA). *P < 0.05, **P < 0.01, and ***P < 0.001 were considered as statistically significant differences; n. s., not significant differences.

## Electronic supplementary material

Below is the link to the electronic supplementary material.

Supplementary Figures

Supplementary Tables

Supplementary Information (full-length western blots)

## Data Availability

Microarray data have been deposited in the Gene Expression Omnibus (GEO) database under accession numbers GSE254887 (HMECs) and GSE283321 (PrECs). For inquiries regarding the data, please contact Akihito Inoko, Aichi Medical University, at inoko.akihito.288@mail.aichi-med-u.ac.jp.
