# Supplementary material for: Long-term expansion of basal cells and the novel differentiation methods identify mechanisms for switching Claudin expression in normal epithelia
Source: Sci Rep. 2025 Apr 9;15:12172. doi: 10.1038/s41598-025-95463-3 (PMC11982363; doi:10.1038/s41598-025-95463-3)
Supplement: Supplementary file 1 — Supplementary Figures [file 41598_2025_95463_MOESM1_ESM.pdf]

Supplementary Figures

Long-term expansion of basal cells and the novel differentiation methods identify mechanisms for switching Claudin expression in normal epithelia

Akihito Inoko[1,*], Norihito Soga[1,2], Minako Suzuki[1], Tohru Kiyono[3], Junichi Ikenouchi[4], Takahiro Kojima[2], Yoshikatsu Sato[5], Daisuke Saito[4], Tatsuo Miyamoto[6], Naoki Goshima[7], Hideaki Ito[1], and Kenji Kasai[1]

[1]Department of Pathology, Aichi Medical University School of Medicine, Nagakute 480-1195, Japan

[2]Department of Urology, Aichi Cancer Center Hospital, Nagoya 464-8681, Japan

[3]Project for Prevention of HPV-Related Cancer, Exploratory Oncology Research and Clinical Trial Center, National Cancer Center, Chiba 277-8577, Japan

[4]Department of Biology, Faculty of Sciences, Kyushu University, Fukuoka, 819-0395, Japan

[5]Institute of Transformative Bio-Molecules (WPI-ITbM), Nagoya University, Nagoya 464-8601, Aichi, Japan

[6]Department of Molecular and Cellular Physiology, Yamaguchi University Graduate School of Medicine, Ube 755-8505, Japan

[7]Molecular Profiling Research Center for Drug Discovery, National Institute of Advanced Industrial Science and Technology, Tokyo 135-0064, Japan

[*]Correspondence should be addressed to Akihito Inoko, M.D., Ph.D.
Department of Pathology, Aichi Medical University School of Medicine
1-1 Yazakokarimata, Nagakute, Aichi 480-1195, Japan
E-mail: inoko.akihito.288@mail.aichi-med-u.ac.jp
Phone +81-561-62-3311, Fax +81-561-61-2350
ORCID 0000-0002-6739-2948

The PDF file includes

Supplementary Figure 1 | Biological replicates with donor 2 and additional details for Figure 1.

Supplementary Figure 2 | Biological replicates with donor 2 and additional data for Figure 3.

Supplementary Figure 3 | Biological replicates with donor 2 and additional data for Figure 4.

Supplementary Figure 4 | Biological replicates with donor 2 and additional data for Figure 5.

Supplementary Figure 1. Biological replicates with donor 2 and additional details for Figure 1.

(A-D) Biological replicates of data in Figure 1, A, C, D, and E (donor 1) with independent donor 2. **(E)** Wide-field images of Figure 1 D plus an image of YDA-depleted cells extending the culture period up to 5 days. The extension did not improve the degree of differentiation. **(F)** P63α antibody used for this research showed a single band at a molecular weight of 72 kDa in immunoblotting (donor 1), suggesting the recognition of ΔNp63α as per a previous report [35]. Abbreviations of inhibitors: Y, 10 μM Y-27632; D, 1 μM DMH1; A, 1 μM A-83-01; and C,1 μM CHIR99021. Scale bars, 100 μm, 10 μm, and 10 μm in B, C, and E, respectively.

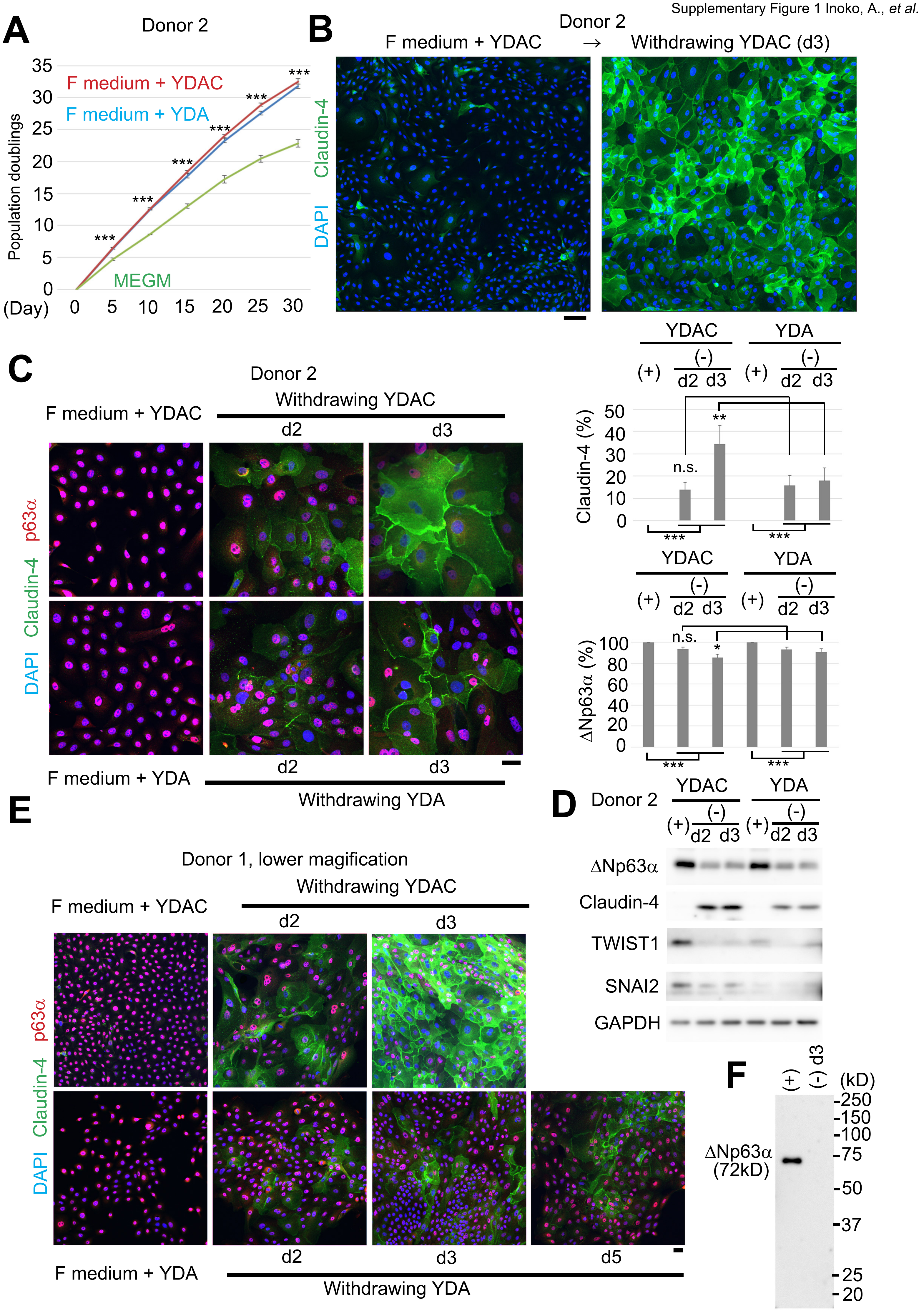
A
Donor 2
F medium + YDAC
F medium + YDA
MEGM
Population doublings
(Day)
***
B
Donor 2
F medium + YDAC
→
Withdrawing YDAC (d3)
Claudin-4
DAPI
C
Donor 2
F medium + YDAC
Withdrawing YDAC
d2
d3
p63α
Claudin-4
DAPI
F medium + YDA
Withdrawing YDA
YDAC
YDA
(+)
(-)
Claudin-4 (%)
n.s.
**
ΔNp63α (%)
*
D
Donor 2
ΔNp63α
Claudin-4
TWIST1
SNAI2
GAPDH
E
Donor 1, lower magification
d5
F
(kD)
ΔNp63α
(72kD)
250
150
100
75
50
37
25
20

Supplementary Figure 2. Biological replicates with donor 2 and additional data for Figure 3.

(A-E) Biological replicates of data in Figure 3 B–E (donor 1) with independent donor 2. **(F)** YDAC-maintained HMECs in Matrigel form well-polarized cystic structures on day 8 with partial expression of EGR1 and ELF3, suggesting heterogeneity in differentiation (immunostaining, arrows). Details are provided in the Materials and Methods section. **(G)** Phase-contrast micrograph of our new method for culturing mammary cells in an in vivo-like bilayer. The bilayer was assembled by culturing cells in the YDAC medium for approximately 3 days after they reached confluence. Cells on the first day of confluence (d1) were compressed and partially layered, and eventually bilayered (d2-3). Bilayering can be estimated based on the intersection of two types of cell-cell boundaries (d3-). See also Figure 3 H. Scale bars, 10 μm.

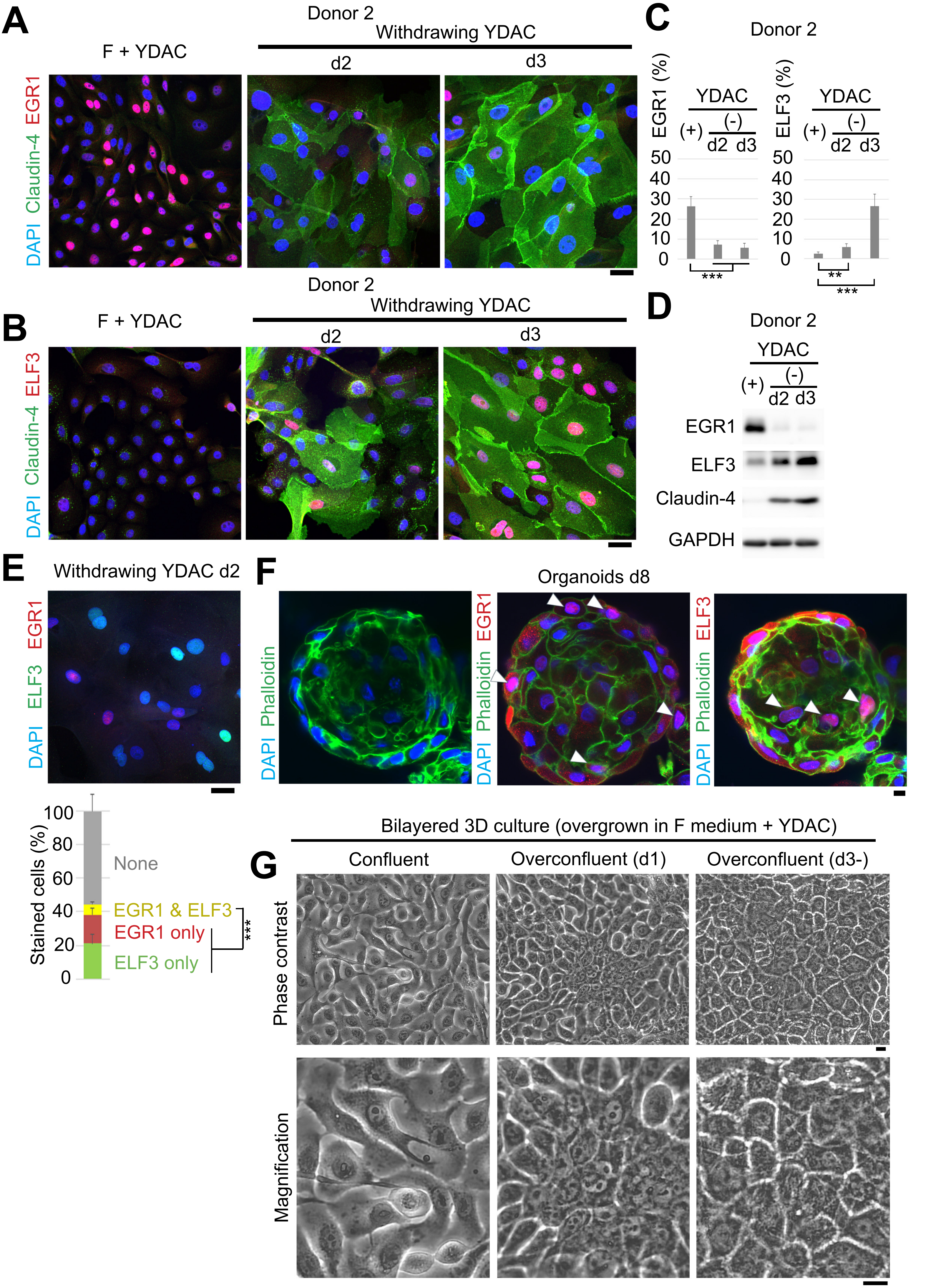

A
Donor 2
Withdrawing YDAC
F + YDAC
d2
d3
DAPI Claudin-4 EGR1
C
Donor 2
EGR1 (%)
ELF3 (%)
YDAC
(+)
(-)
d2 d3
50
40
30
20
10
0
***
**
***
B
Donor 2
Withdrawing YDAC
F + YDAC
d2
d3
DAPI Claudin-4 ELF3
D
Donor 2
YDAC
(+)
(-)
d2 d3
EGR1
ELF3
Claudin-4
GAPDH
E
Withdrawing YDAC d2
DAPI ELF3 EGR1
Stained cells (%)
100
80
60
40
20
0
None
EGR1 & ELF3
EGR1 only
ELF3 only
***
F
Organoids d8
DAPI Phalloidin
DAPI Phalloidin EGR1
DAPI Phalloidin ELF3
Bilayered 3D culture (overgrown in F medium + YDAC)
G
Confluent
Overconfluent (d1)
Overconfluent (d3-)
Phase contrast
Magnification

Supplementary Figure 3. Biological replicates with donor 2 and additional data for Figure 4.

(A and B) Biological replicates of data in Figure 4 A and B (donor 1) with independent donor 2. **(C)** Left, phase-contrast images of siEGR1-treated organoids from independent donors. Right, quantification of the organoids larger than 80 μm in diameter. Fifty organoids were counted in each group (n=3). Scale bars, 10 μm and 100 μm, in A and C, respectively.

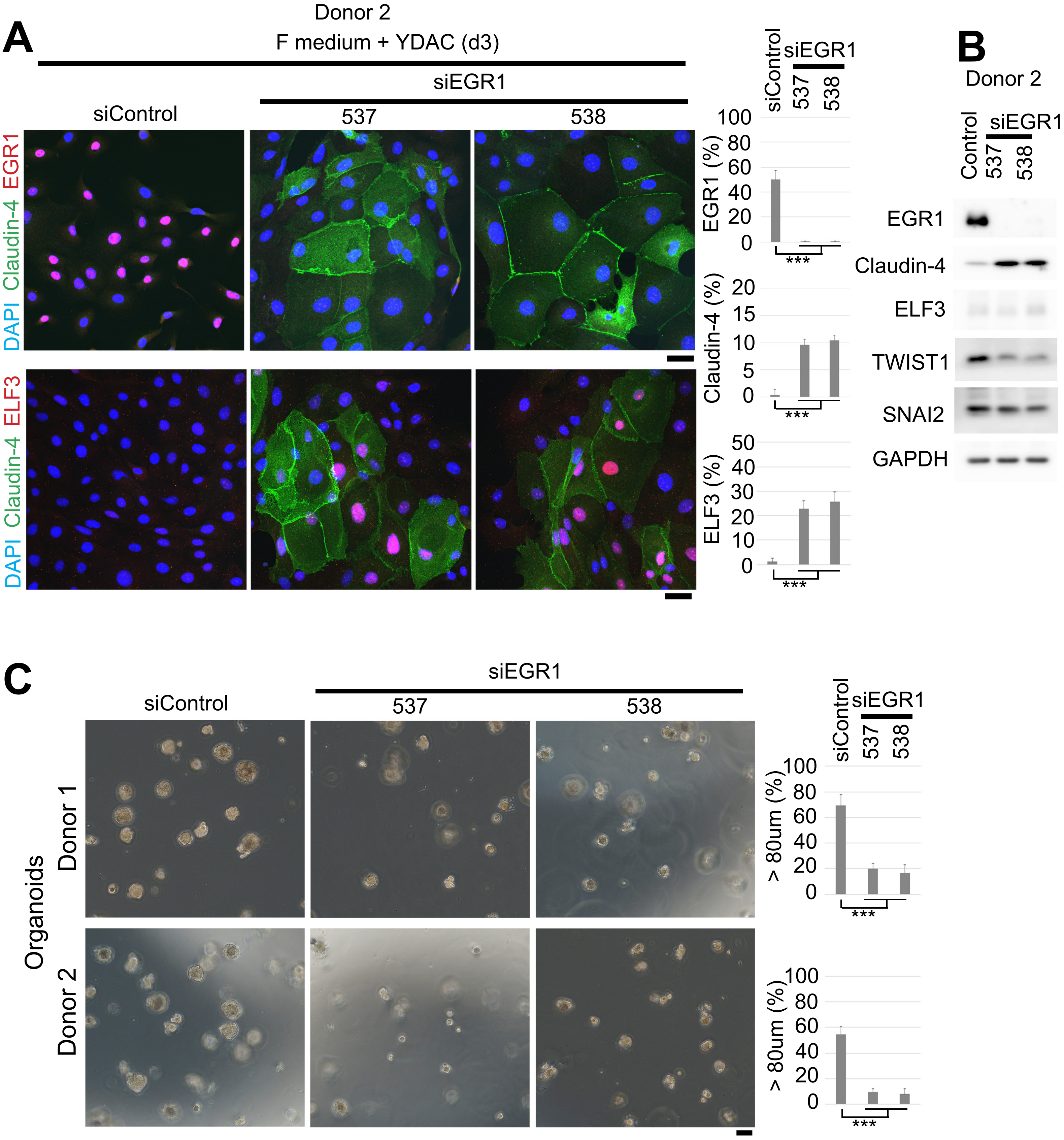
A
Donor 2
F medium + YDAC (d3)
siEGR1
siControl
537
538
DAPI Claudin-4 EGR1
DAPI Claudin-4 ELF3
siControl
siEGR1
537
538
EGR1 (%)
100
80
60
40
20
0
***
Claudin-4 (%)
20
15
10
5
0
***
ELF3 (%)
50
40
30
20
10
0
***
B
Donor 2
Control
siEGR1
537
538
EGR1
Claudin-4
ELF3
TWIST1
SNAI2
GAPDH
C
siEGR1
siControl
537
538
Organoids
Donor 1
Donor 2
siControl
siEGR1
537
538
> 80um (%)
100
80
60
40
20
0
***
> 80um (%)
100
80
60
40
20
0
***

Supplementary Figure 4. Biological replicates with donor 2 and additional data for Figure 5.

(A-C) Biological replicates of Figure 5 A-C (donor 1) with independent donor 2. **(D)** Left, phase-contrast images of siELF3-treated organoids from independent donors. Right, quantification of the organoids with a bumpy shape. Fifty organoids were counted in each group (n=3). Scale bars, 10 μm, 10 μm, and 100 μm, in A, C, and D, respectively.

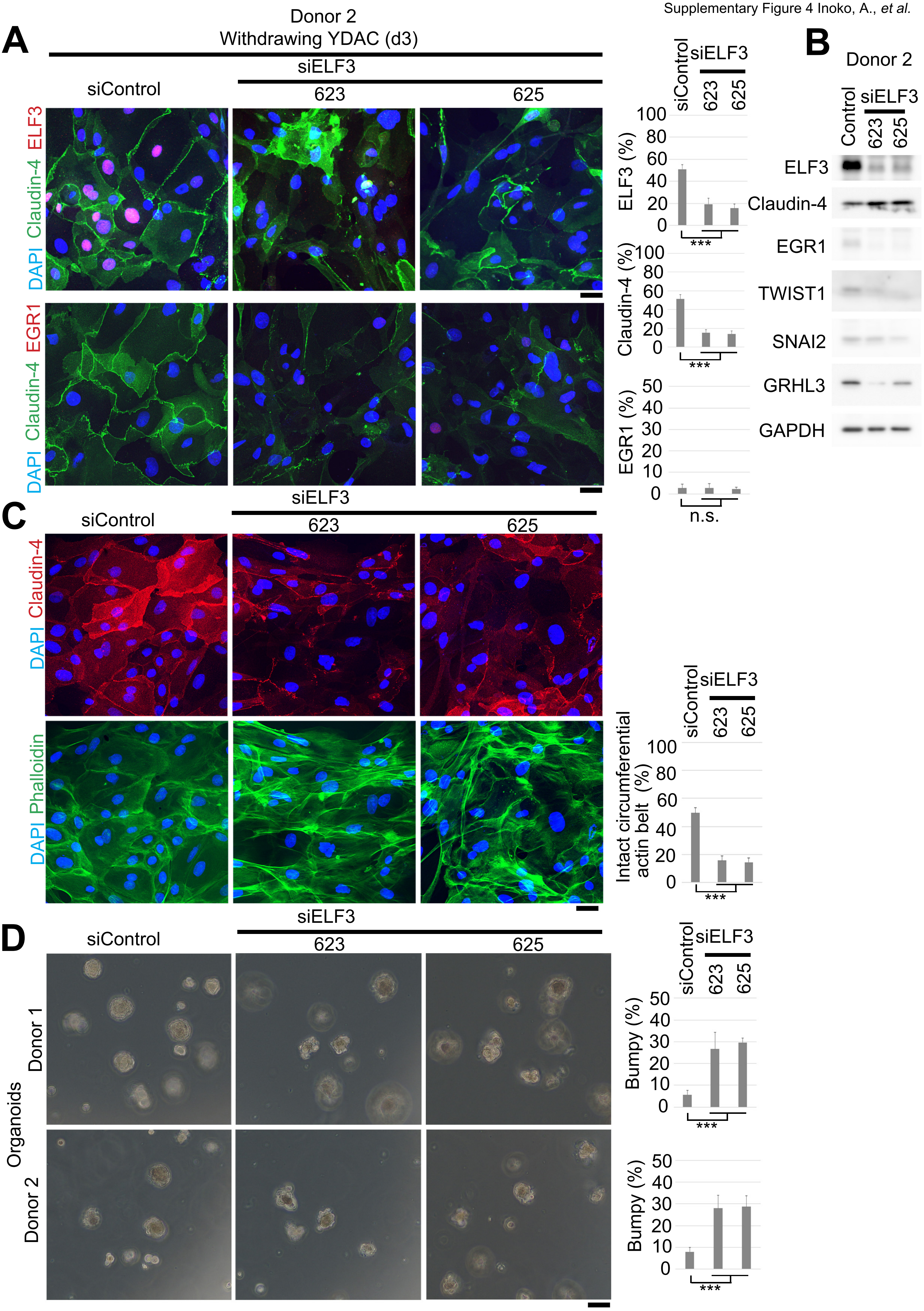
A
Donor 2
Withdrawing YDAC (d3)
siELF3
siControl
623
625
DAPI Claudin-4 ELF3
DAPI Claudin-4 EGR1
siControl
siELF3
623
625
ELF3 (%)
100
80
60
40
20
0
***
Claudin-4 (%)
100
80
60
40
20
0
***
EGR1 (%)
50
40
30
20
10
0
n.s.
B
Donor 2
Control
siELF3
623
625
ELF3
Claudin-4
EGR1
TWIST1
SNAI2
GRHL3
GAPDH
C
siControl
siELF3
623
625
DAPI Claudin-4
DAPI Phalloidin
siControl
siELF3
623
625
Intact circumferential actin belt (%)
100
80
60
40
20
0
***
D
siControl
siELF3
623
625
Organoids
Donor 1
Donor 2
siControl
siELF3
623
625
Bumpy (%)
50
40
30
20
10
0
***
Bumpy (%)
50
40
30
20
10
0
***
