# Supplementary material for: Long-term expansion of basal cells and the novel differentiation methods identify mechanisms for switching Claudin expression in normal epithelia
Source: Sci Rep. 2025 Apr 9;15:12172. doi: 10.1038/s41598-025-95463-3 (PMC11982363; doi:10.1038/s41598-025-95463-3)

Inoko, A., *et al.*

Supplementary Information

Full-length western blots and overlaid membranes with molecular weight markers

Figure 1 E

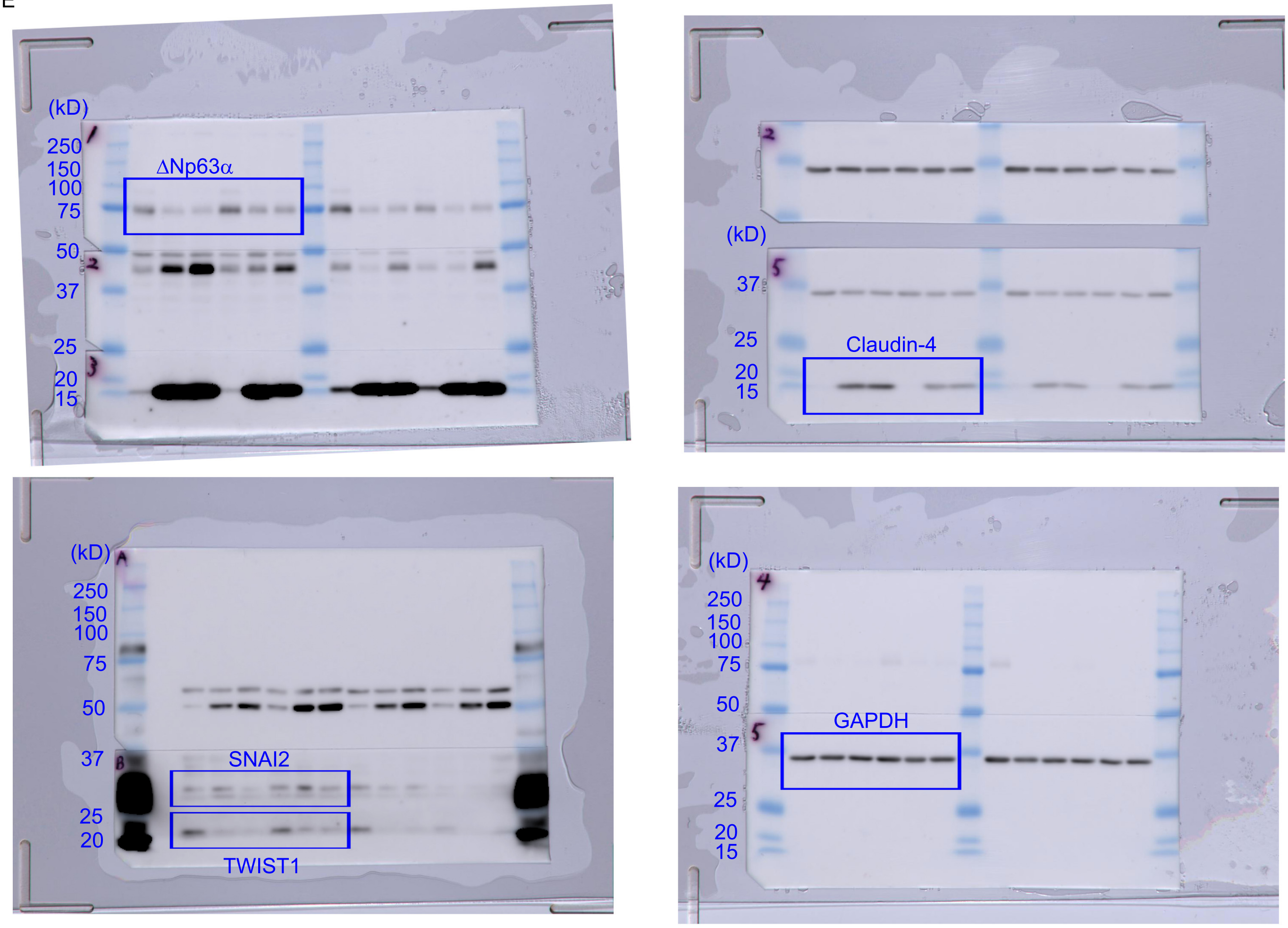

Supplementary Figure 1 D

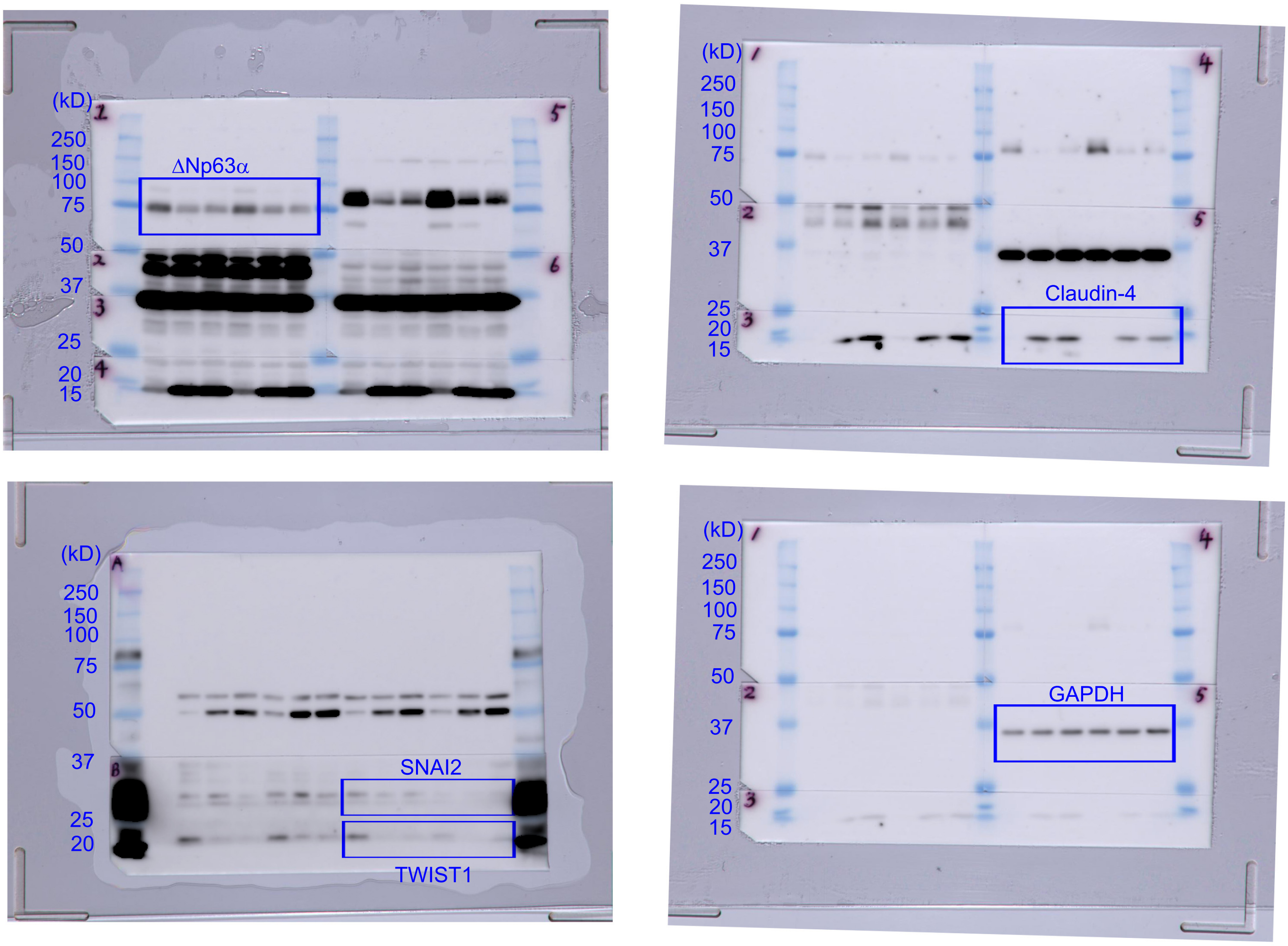

Figure 2 C

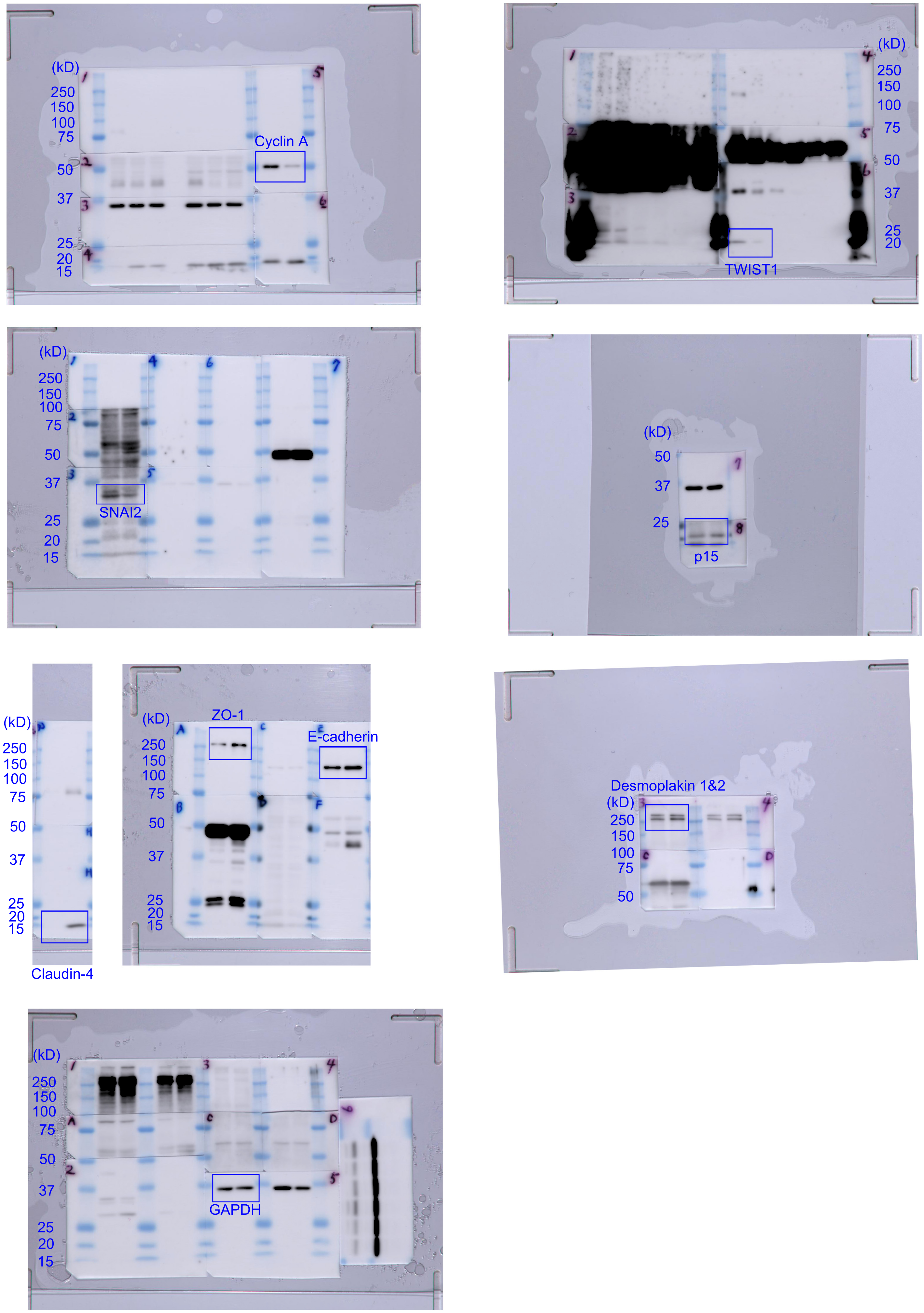

(kD)
250
150
100
75
50
37
25
20
15
Cyclin A
TWIST1
SNAI2
p15
Claudin-4
ZO-1
E-cadherin
Desmoplakin 1&2
GAPDH

Figure 2 D

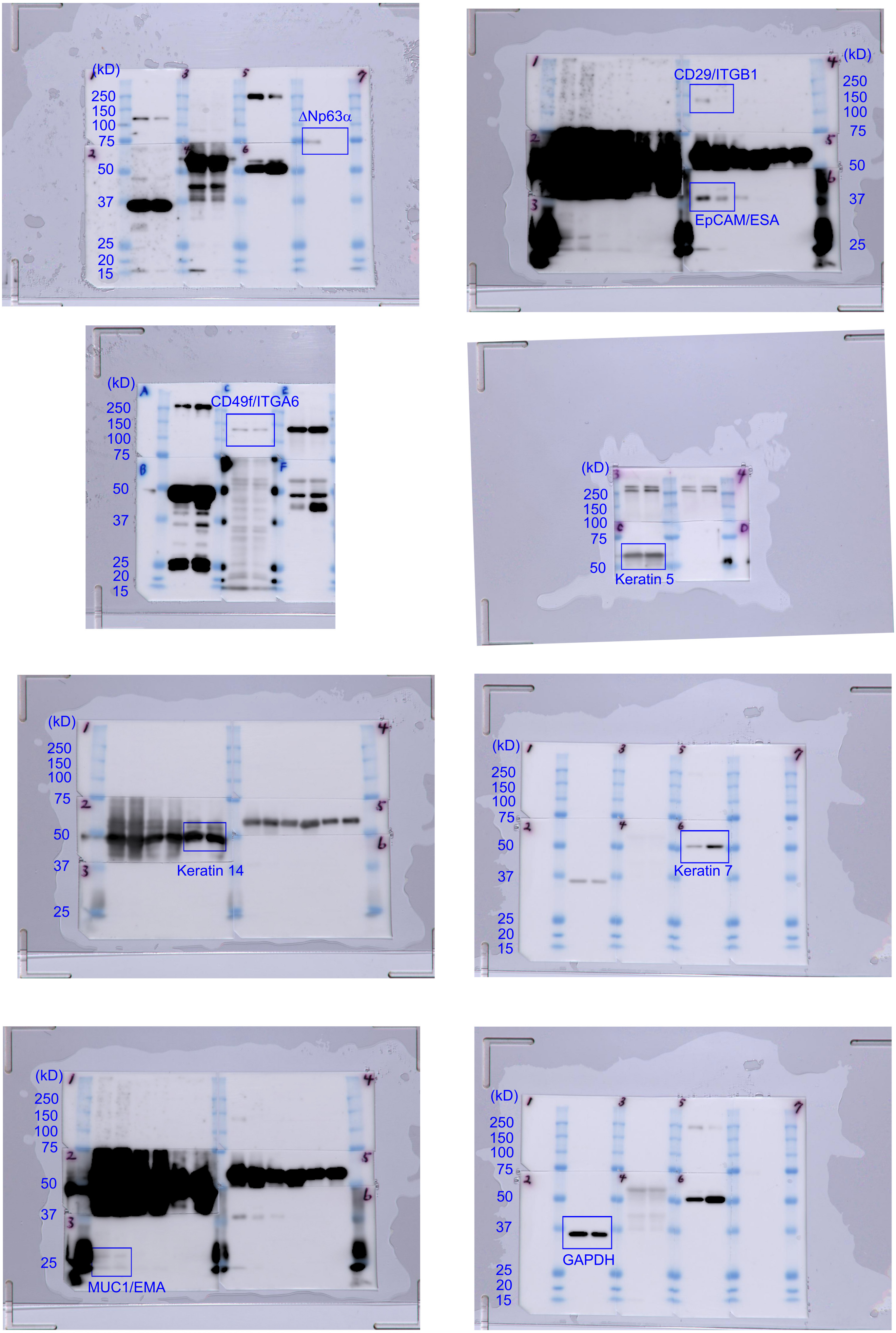

(kD)
250
150
100
75
50
37
25
20
15
ΔNp63α
CD29/ITGB1
EpCAM/ESA
CD49f/ITGA6
Keratin 5
Keratin 14
Keratin 7
MUC1/EMA
GAPDH

Figure 3 E

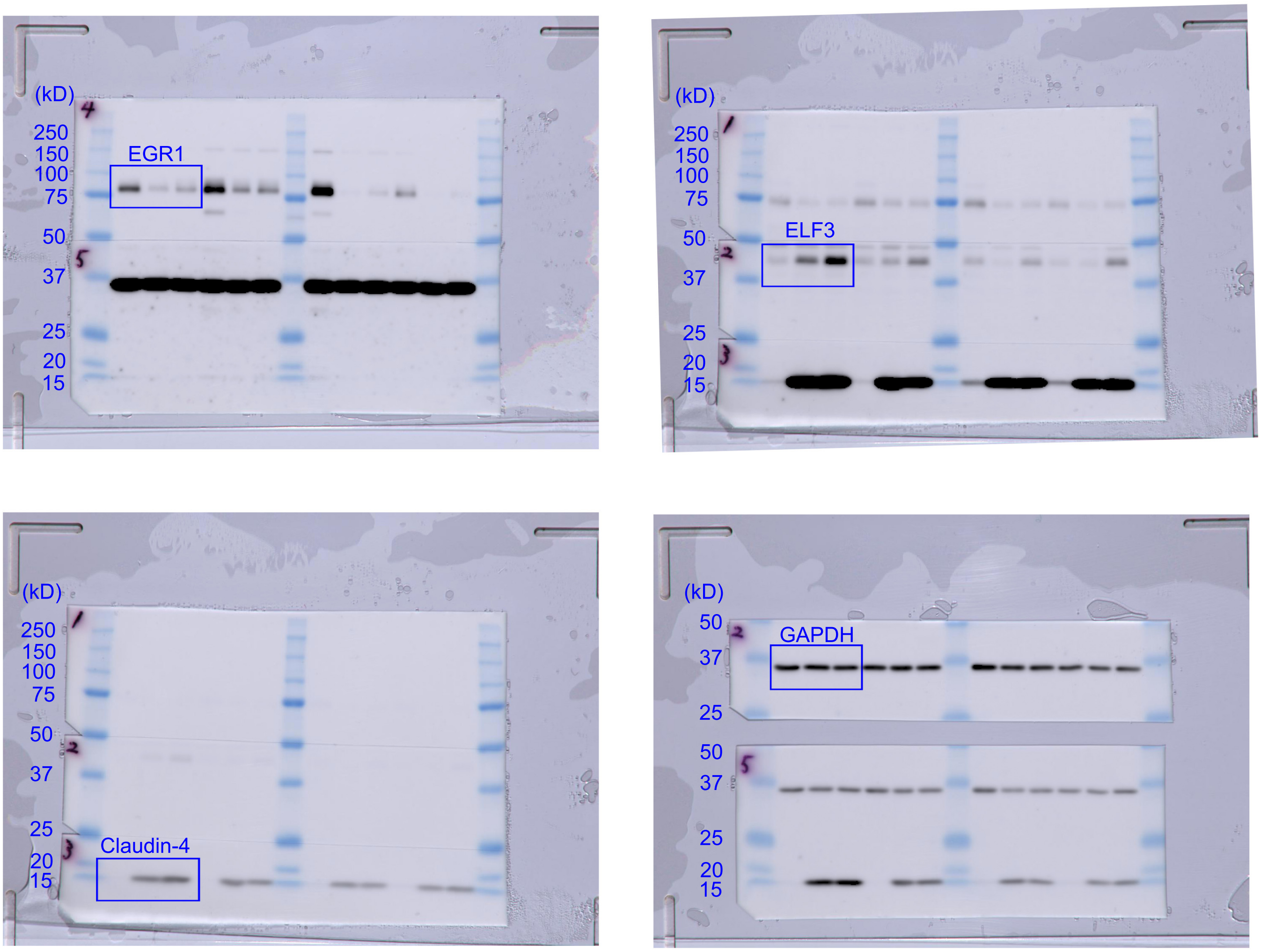

Supplementary Figure 2 D

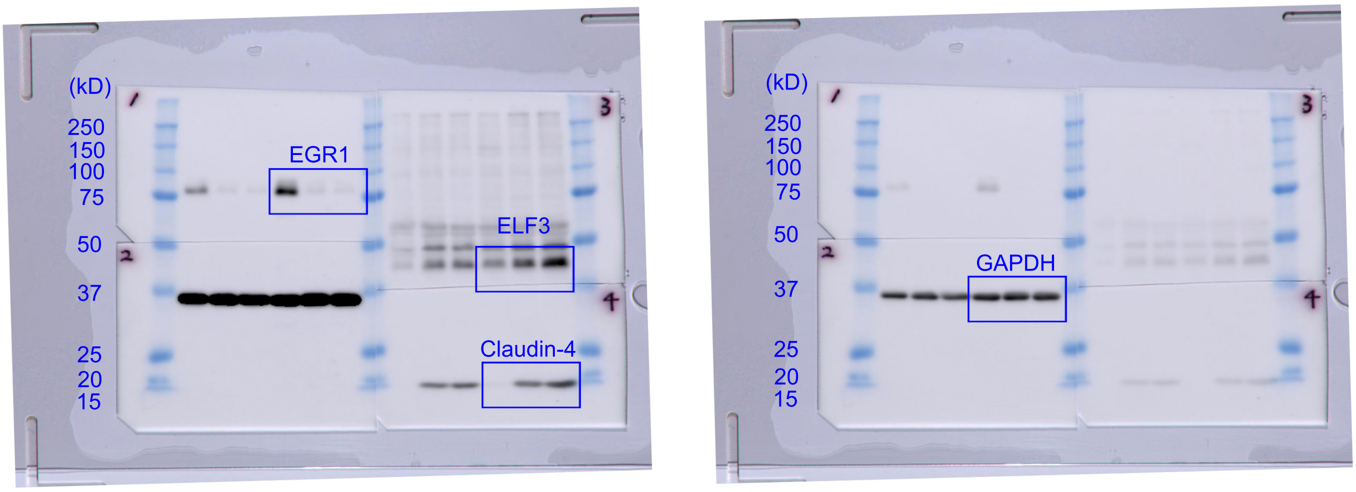

Figure 4 B

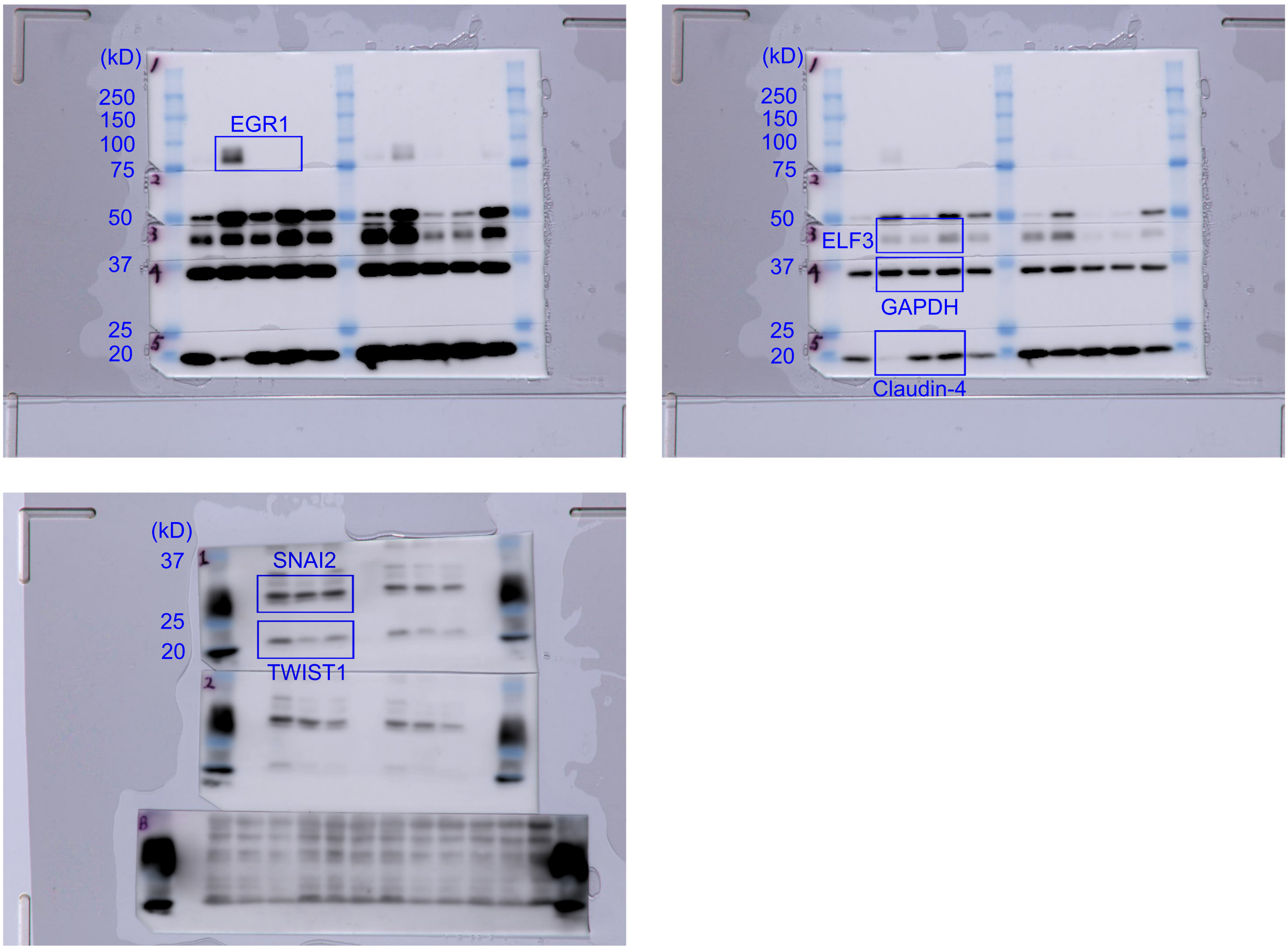

Figure 4 D

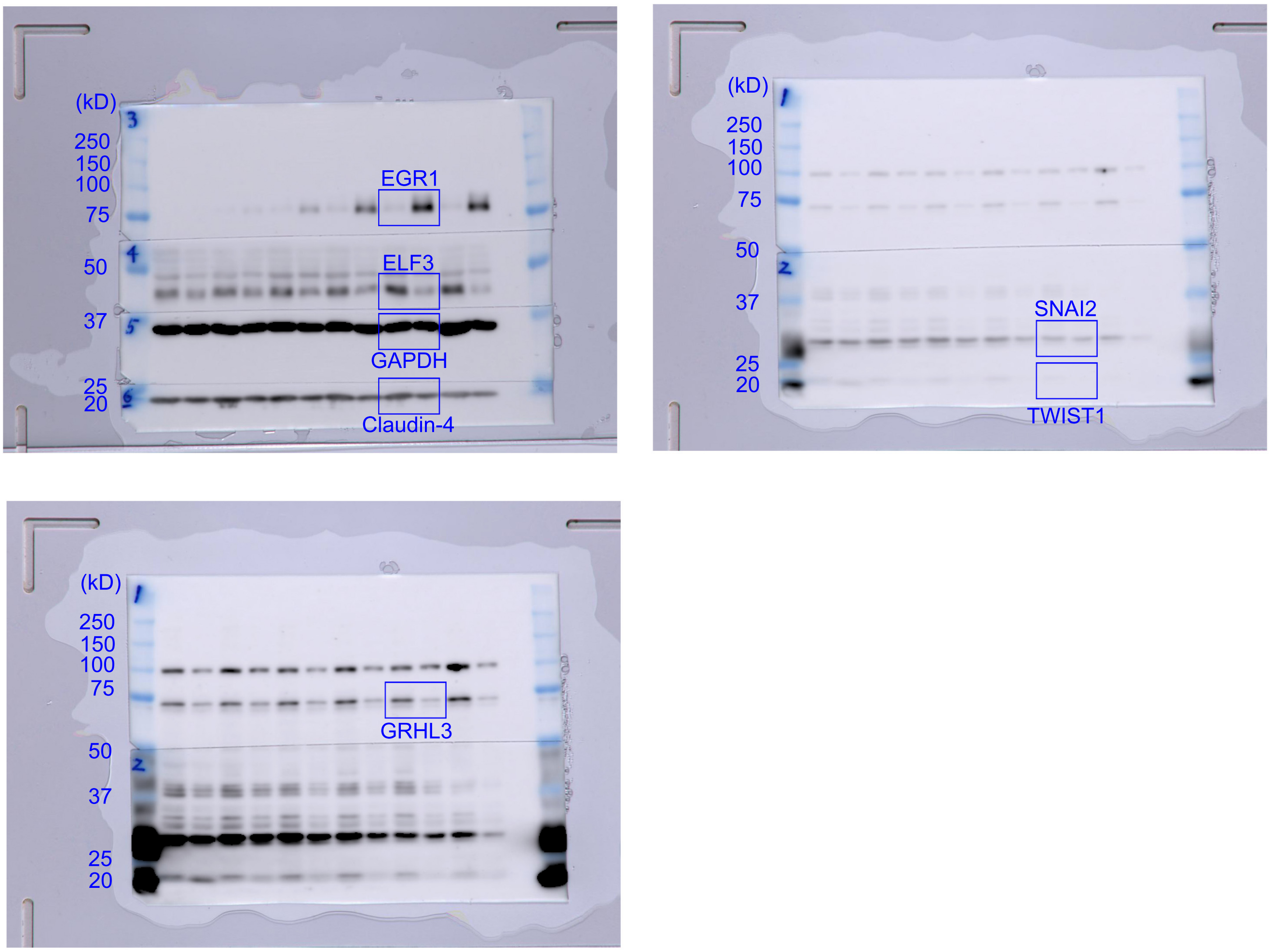

Supplementary Figure 3 B

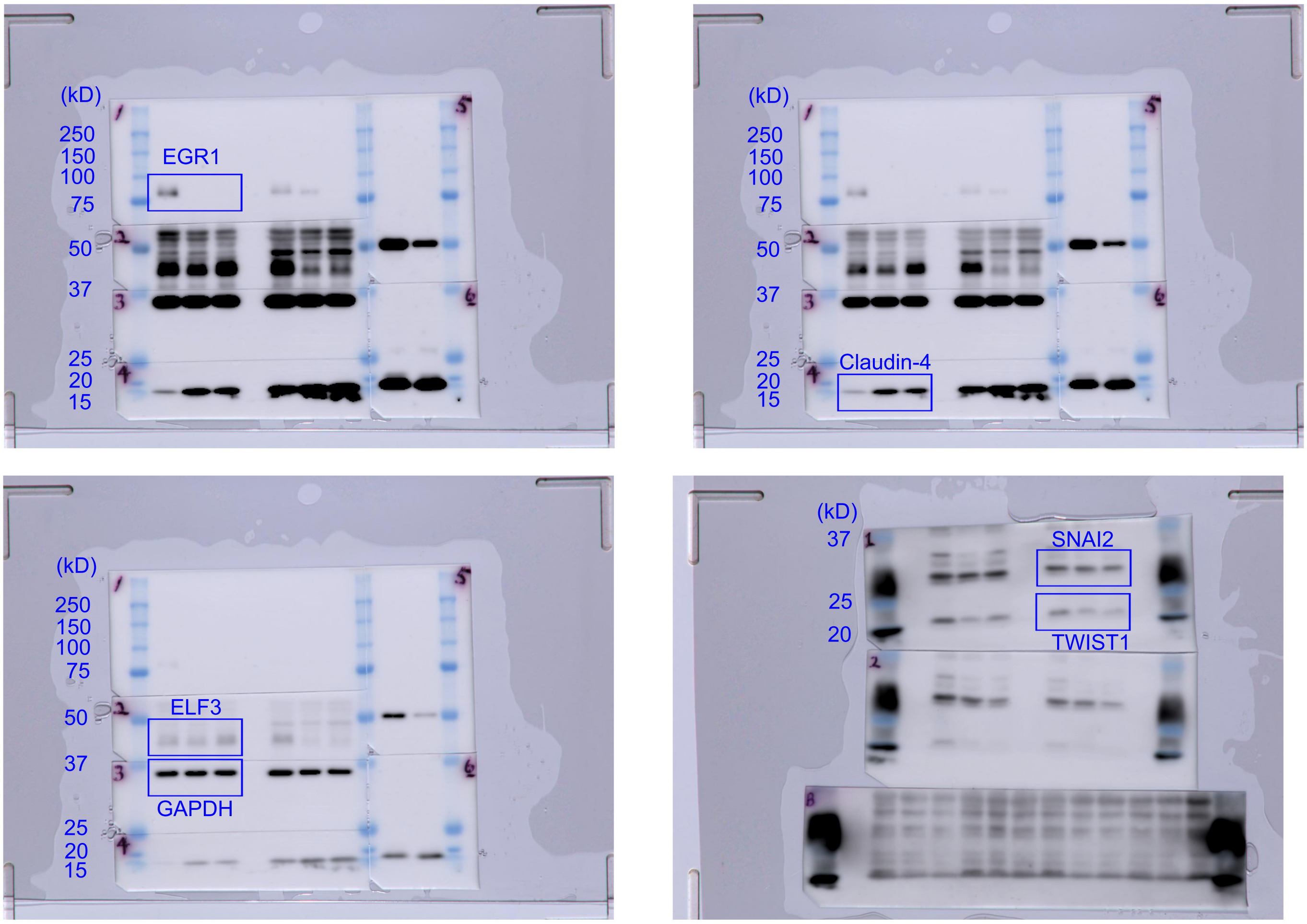

Figure 5 B

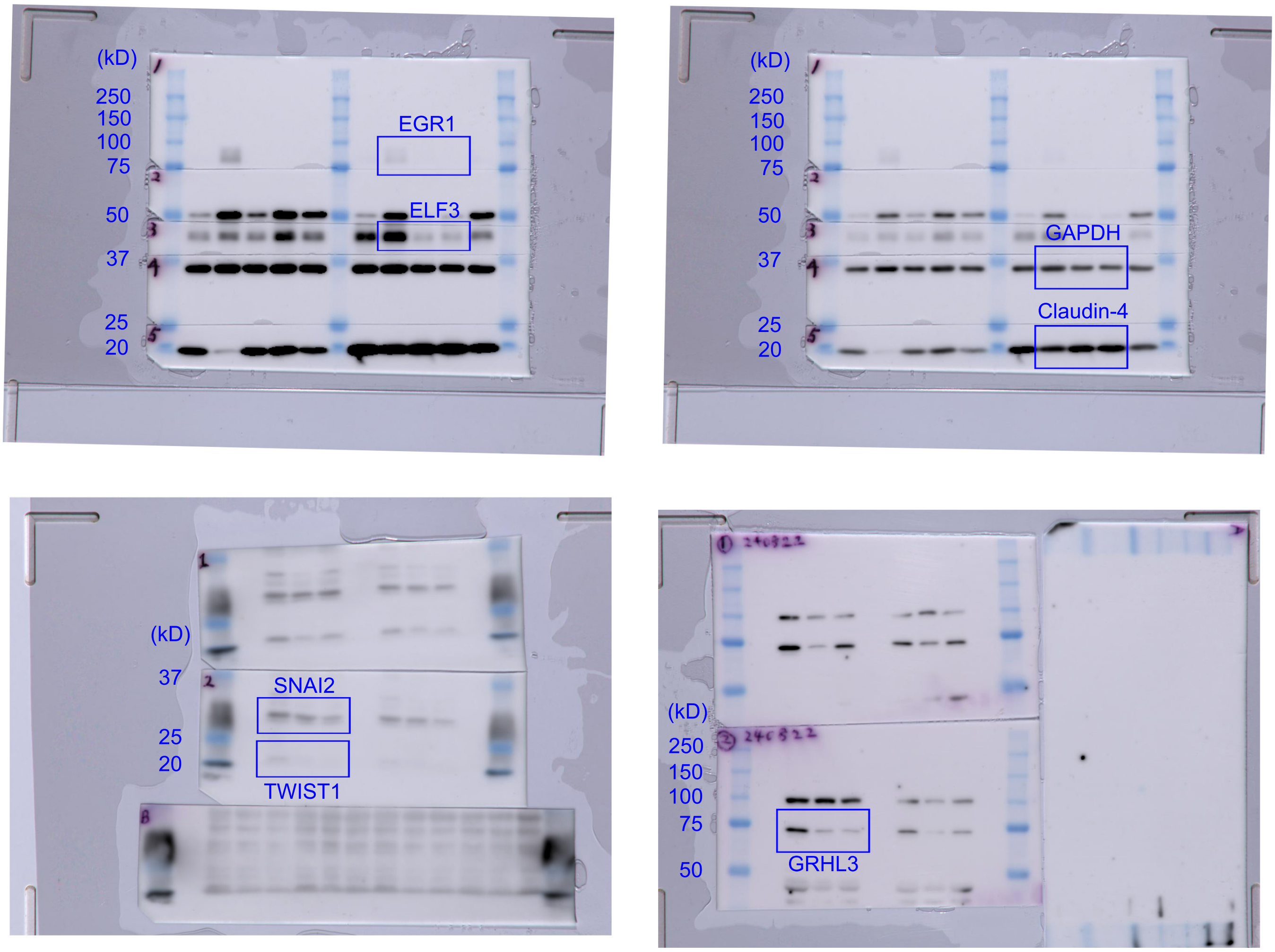

Figure 5 E

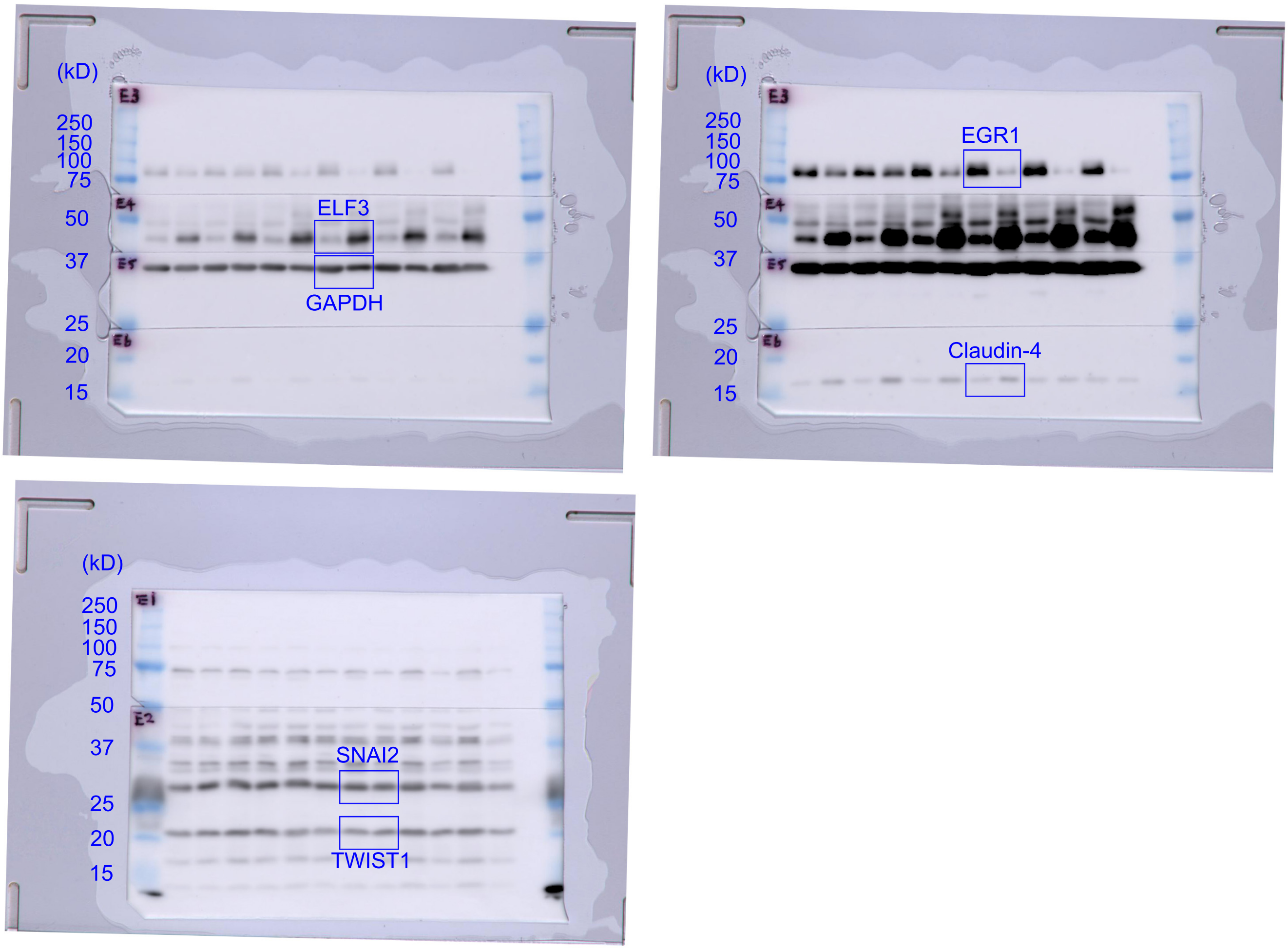

Supplementary Figure 4 B

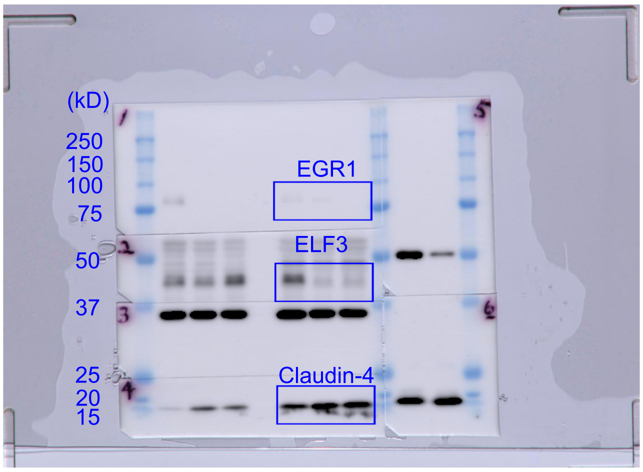

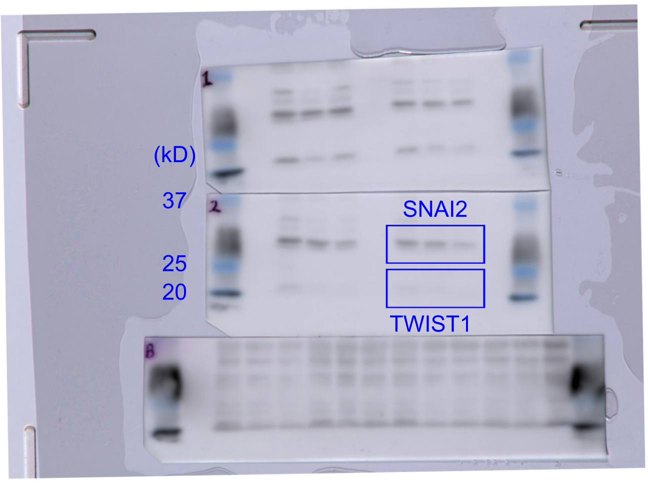

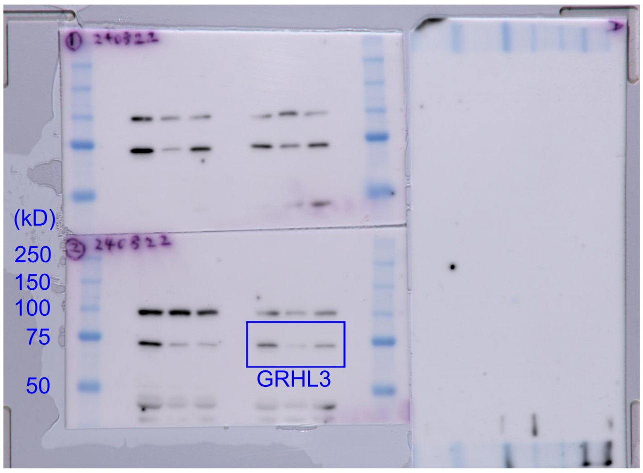

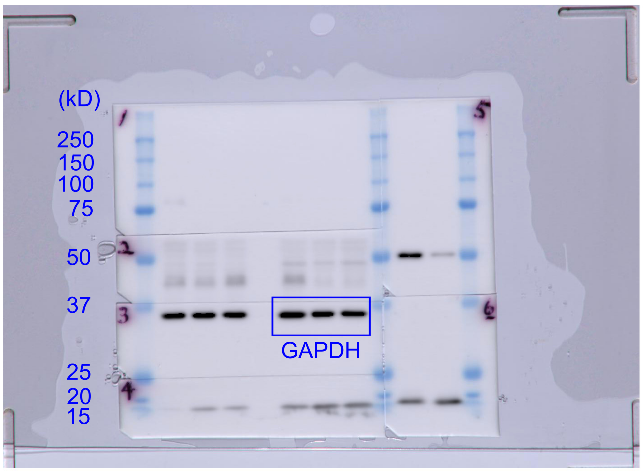

Supplement: Supplementary file 3 — Supplementary Information (full-length western blots) [file 41598_2025_95463_MOESM3_ESM.pdf]
